# Structural diversity among *Acinetobacter baumannii* K-antigens and its implication in the *in silico* serotyping

**DOI:** 10.3389/fmicb.2023.1191542

**Published:** 2023-06-21

**Authors:** Janardhanaachari Roshini, L. Ponoop Prasad Patro, Sruthi Sundaresan, Thenmalarchelvi Rathinavelan

**Affiliations:** Department of Biotechnology, Indian Institute of Technology Hyderabad, Kandi, Telangana, India

**Keywords:** K-antigen structure, capsular polysaccharide, antimicrobial resistance, K-typing, Wzx/Wzy-dependent pathway, *Acinetobacter baumannii*

## Abstract

*Acinetobacter baumannii* is an emerging opportunistic pathogen. It exhibits multi-, extreme-, and pan-drug resistance against several classes of antibiotics. Capsular polysaccharide (CPS or K-antigen) is one of the major virulence factors which aids *A. baumannii* in evading the host immune system. K-antigens of *A. baumannii* exploit the Wzx/Wzy-dependent pathway that involves 13 different proteins for its assembly and transport onto the outer membrane. A total of 64 (out of 237 K-locus(KL) types) known K-antigen sugar repeating structures are discussed here and are classified into seven groups based on their initial sugars, QuiNAc4NAc, GalNAc, GlcNAc, Gal, QuiNAc/FucNAc, FucNAc, and GlcNAc along with Leg5Ac7Ac/Leg5Ac7R. Thus, the corresponding seven initializing glycosyltransferases (ItrA1, ItrA2, ItrA3, ItrA4, ItrB1, ItrB3, and ItrA3 along with ItrB2) exhibit serotype specificity. The modeled 3D-structural repository of the 64 K-antigens can be accessed at https://project.iith.ac.in/ABSD/k_antigen.html. The topology of K-antigens further reveals the presence of 2-6 and 0-4 sugar monomers in the main and side chains, respectively. The presence of negatively (predominant) or neutrally charged K-antigens is observed in *A. baumannii*. Such diversity in the K-antigen sugar composition provides the K-typing specificity (*viz*., 18–69% in terms of reliability) for Wza, Wzb, Wzc, Wzx, and Wzy proteins involved in the Wzx/Wzy-dependent pathway. Interestingly, the degree of uniqueness of these proteins among different K-types is estimated to be 76.79%, considering the 237 reference sequences. This article summarizes the *A. baumannii* K-antigen structural diversity and creation of a K-antigen digital repository and provides a systematic analysis of the K-antigen assembly and transportation marker proteins.

## Importance

The World Health Organization (WHO) has listed *A. baumannii* as one of the critical pathogens which are in urgent need of new antibiotics. One of the virulence factors responsible for the pathogenicity of *A. baumannii* is the capsular polysaccharides (alternatively, K-antigens). Here, the structural and topological diversity of 64 *A. baumannii* K-antigens is analyzed, modeled, and classified. These models have been deposited in a 3D-structural repository. This structural information will be helpful in therapeutic treatment, vaccine development, phage therapy, and understanding the host–pathogen interactions. Effective serotyping using only the K-antigen marker proteins is discussed in view of outbreak surveillance.

## Introduction

*Acinetobacter* species are aerobic Gram-negative coccobacilli (Peleg et al., 2008) whose genus consists of more than 50 species (Wong et al., 2017), among which *Acinetobacter baumannii* is the most important species in causing infections in humans. *A. baumannii* is an opportunistic nosocomial pathogen responsible for major hospital-acquired diseases such as septicemia, meningitis, and urinary tract and wound infections (Monem et al., 2020). They mainly target immune-compromised patients (Monem et al., 2020) or patients undergoing chemotherapy, transplantation, *etc*. It is also characterized as the most common cause of ventilator-associated pneumonia in several hospitals in ICUs across the world (Ciginskiene et al., 2019). *A. baumannii* is also responsible for causing 8.4% of ventilator-associated pneumonia (VAP) and 2.3% of infections caused by central line-associated bloodstream infections in the USA. In total, 65% of pneumonia cases reported in the USA and Europe are because of carbapenem-resistant *Acinetobacter baumannii* (CRAB) (Kim et al., 2014). Thus, it is a clinically well-studied and characterized species (Harding et al., 2018).

*Acinetobacter baumannii* belongs to a group of pathogens called *ESKAPE* (which stands for *Enterococcus faecium, Staphylococcus aureus, Klebsiella pneumoniae, Acinetobacter baumannii, Pseudomonas aeruginosa*, and *Enterobacter* spp.), as they can easily evade the antibiotic treatment. *A. baumannii* recruits several mechanisms and exhibits various levels of resistance against several classes of antibiotics. The multidrug-resistant (MDR) *A. baumannii* is the most widely spread MDR pathogen in recent times (Geisinger et al., 2019). Apart from being a pathogen responsible for MDR, *A.baumannii* is also associated with extreme drug resistance (XDR) and pan-drug resistance (PDR) (Vrancianu et al., 2020). Furthermore, frequent outbreaks of multidrug-resistant *A. baumannii* strains are of major concern (Qu et al., 2016; Cornejo-Juarez et al., 2020; Kurihara et al., 2020; Brasiliense et al., 2021). Thus, *A. baumannii* is listed as one of the critical pathogens in the World Health Organization's (WHO) priority pathogens list, for which new antibiotics are urgently needed (World Health Organization, 2017).

Several virulence factors are responsible for inducing pathogenicity by *A. baumannii*. These include capsular polysaccharides (CPS), lipooligosaccharides (LOS), outer membrane protein (OMP) (Wong et al., 2017), and type IV pili (Harding et al., 2018). Among them, membrane polysaccharides, LOS and CPS, are utilized in the serotyping of *A. baumannii*. Serotyping plays a vital role during outbreak surveillance as each serotype differs by its chemical makeup, and the nature of infection may also be different. Thus, the effect of antibiotics may vary between serotypes, and different serotypes may have different responses to vaccines (Kamuyu et al., 2021). However, the surface of *A. baumannii* lacks the conventional O-antigen attached to lipid A, unlike in many other Gram-negative bacteria (Kinsella et al., 2015). Indeed, it has been shown that *A. baumannii* can exist without LOS (Simpson et al., 2021) but with the support of other carbohydrate structures (Simpson et al., 2021). Thus, CPS is emphasized in the current investigation. The CPS, also known as K-antigen, is the major virulent factor of *A. baumannii*, which helps in biofilm formation and evades the host immune system, thereby protecting the bacteria from harsh environments (Russo et al., 2010). The CPS follows the Wzx/Wzy-dependent pathway for its assembly and transport. It typically consists of four to six sugar units unique to each serotype, which acts as a scaffold for its multimerization and growth. Several proteins are involved in the CPS repeating unit assembly, multimerization, and surface exportation.

Since the CPS (or K-antigen) (Campos et al., 2004; Llobet et al., 2008; Sachdeva et al., 2017) and LPS (lipopolysaccharide or O-antigen) (Yethon and Whitfield, 2001; Matsuura, 2013; Zhang et al., 2013; Maldonado et al., 2016) surface antigens of Gram-negative bacteria play vital roles in therapeutic treatment, vaccine development, phage therapy, and understanding host–pathogen interactions, the knowledge about their three-dimensional structures is of major importance. Realization of this aspect has led to several time-to-time reviews on K- and O-antigen structures of Gram-negative bacteria such as *E. coli* (Stenutz et al., 2006; Liu et al., 2020), *Salmonella* (Liu et al., 2014), and *Shigella* (Liu et al., 2008).

Due to the upsurge in the immune evasion and multidrug resistance strategies exhibited by *A. baumannii*, there is a need to explore alternative therapeutic options to antibiotics (Shahid et al., 2021). To this end, several attempts have been made to exploit monoclonal antibodies (Russo et al., 2013; Nielsen et al., 2017), whole-cell vaccines (Lopez-Siles et al., 2021), outer membrane complex and bacterial ghost vaccines (Lopez-Siles et al., 2021), polysaccharide-based vaccines (Lopez-Siles et al., 2021), DNA-based vaccines (Lopez-Siles et al., 2021), and protein-based vaccines (Lopez-Siles et al., 2021; McConnell and Martin-Galiano, 2021) toward the prevention of *A. baumannii* infections. For polysaccharide-based vaccines, CPS and LPS can readily be utilized. Indeed, it has been shown that in the preclinical infection models, K1 CPS is immunogenic upon immunization, and opsonophagocytosis of *A. baumannii* is also facilitated *in vitro* by the antibodies against this antigen (Russo et al., 2013). Thus, K1 can be a potential therapeutic target. Interestingly, a recent study has shown that a vaccine with synthetic pseudaminic acid-conjugated carrier protein confers effective protection against *A. baumannii* infection (Wei et al., 2021). Since many of the *A. baumannii* K-antigens (quite heterogenic in their sugar composition) are seen in the clinical samples of hospitalized patients (Supplementary Table S1), it is necessary to develop a polyvalent vaccine that can cover many or all of the K-antigens. Importantly, the sugar composition of the K-antigen is shown to play an essential role in dictating the virulence of the *A. baumannii*, in such a way that the removal of a glycosyl transferase results in the removal of the sugar branch, making it virulent (Talyansky et al., 2021). These necessitate detailed information about the sugar composition, linkage, and stereoisomeric (enantiomeric and epimeric) forms of all the *A. baumannii* K-antigens in one place. Furthermore, a thorough understanding of *A. baumannii* CPS diversity is critical in successfully implementing phage therapy to treat the infections caused by the pathogen (Tu et al., 2023). A noteworthy point is that the knowledge about the sugar composition of K3 antigen has recently been shown to produce *A. baumannii* isolate that is susceptible to bacteriophage, as the isolate produced a K3 antigen variant that lacks a branch sugar (Timoshina et al., 2023). In addition, the CPS is suggested to be an obstacle for passive immunization strategies as K1 CPS inhibits the binding of monoclonal antibodies against outer membrane proteins (Wang-Lin et al., 2017). Indeed, the composition (Wei et al., 2021) and 3D structural information of the K-antigen are found to be important (Carboni and Adamo, 2020) as they play crucial roles in binding with antibodies (Ozdilek et al., 2021). These necessitate the details about the sugar composition and three-dimensional structure of all the *A. baumannii* K-antigens to design a vaccine containing antibodies against different K-antigens or having common sugar(s) found in different K-antigens. To this end, 64 known K-antigen structures of *A. baumannii* are presented and discussed here.

## Materials and methods

### 3D-structure modeling of *A. baumannii* K-antigens

Before the modeling of *A. baumannii* K-antigen 3D structures, their chemical formula describing the sugar composition, the linkage between adjacent sugars, and their stereoisomeric forms were collected from the literature (Table 1). The initial models of the K-antigens having regular sugars were built using GLYCAM-Web (Woods, 2005–2023). The K-antigens having unusual sugars were initially modeled using GLYCAM-Web with the closest sugar in place of the unusual sugar, and finally, the appropriate sugar was manually modeled using a PyMOL molecular modeling tool (Schrodinger, 2015). Subsequently, the modeled K-antigens were energy minimized using CHARMM 36 forcefield (Huang and MacKerell, 2013). A negative charge of −1 was considered (due to the presence of a carboxylic group) if a K-antigen was found to have any one of the following sugars: glucuronic acid, galacturonic acid, pyruvic acid, pseudaminic acid, legionaminic acid, or bacillosamine. If more than one of the above sugars were found, the total negative charge was equivalent to the total number of such sugars. This formal charge was then assigned as the charge of the K-antigen during the minimization. Generalized Born with a simple SWitching (GBSW) implicit solvation was used during the minimization. The minimization was performed for 1,500 steps of steepest descent (SD), further followed by 1,500 steps of the adopted basis Newton–Raphson method. Notably, the CHARMM force field is used in the current study to model the K-antigen structures, as it has been widely used for the modeling of carbohydrates and their derivatives as well as to capture their conformational dynamics since 2011 (Guvench et al., 2011).

**Table 1:** Published chemical representation of 64 K-antigens of *A. baumannii*.

**K-antigen**	**Chemical representation**	**Igt**	**Reference**
K1	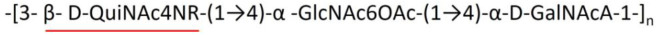	ItrA1	(Russo et al., 2013)
K2	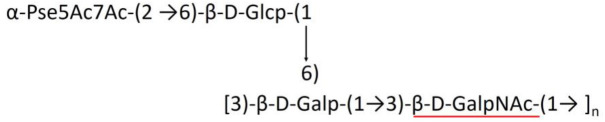	ItrA2	(Kenyon et al., 2014)
K3	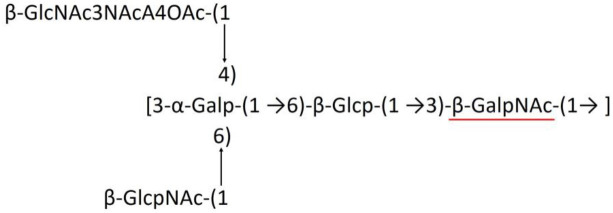	ItrA2	(Singh et al., 2018)
K4	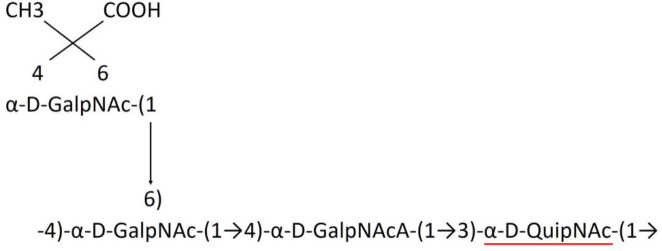	ItrB1	(Kenyon et al., 2016b)
K5	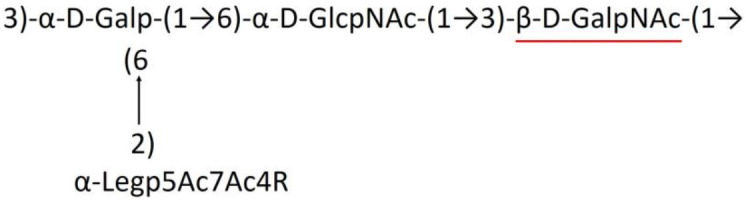	ItrA2	(Kenyon et al., 2019)
K6	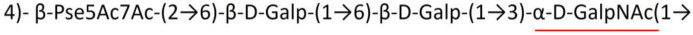	ItrA2	(Kenyon et al., 2015b)
K7	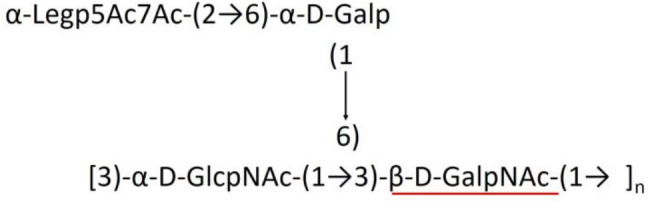	ItrA2	(Senchenkova et al., 2019)
K8	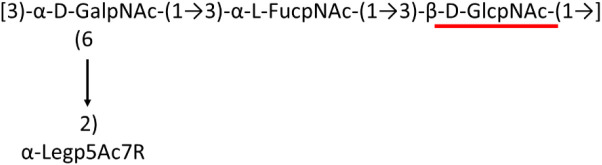	ItrA3; ItrB2	(Arbatsky et al., 2019)
K11	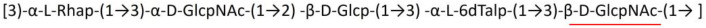	ItrA3	(Kenyon et al., 2017c)
K12	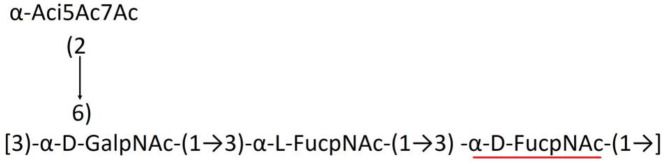	ItrB3	(Kenyon et al., 2015c)
K13	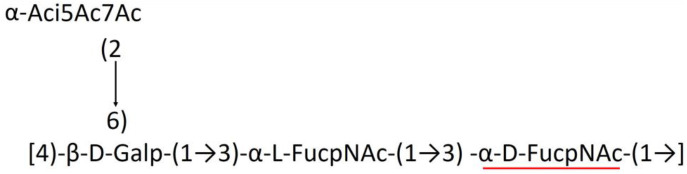	ItrB3	(Kenyon et al., 2017a)
K14	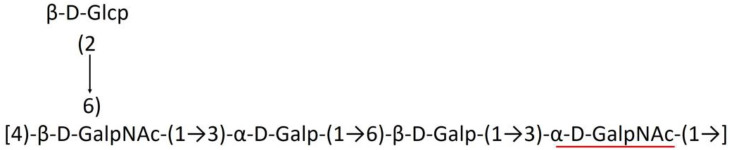	ItrA2	(Kenyon et al., 2015a)
K15	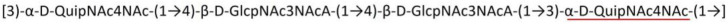	ItrA1	(Shashkov et al., 2017)
K16	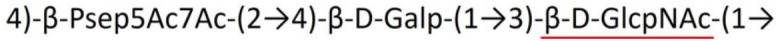	ItrA3	(Kenyon et al., 2019)
K17	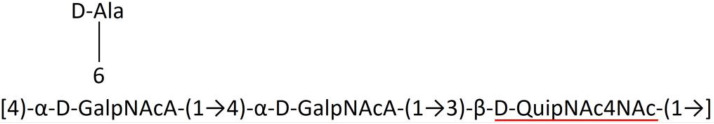	ItrA1	(Kenyon et al., 2020)
K19	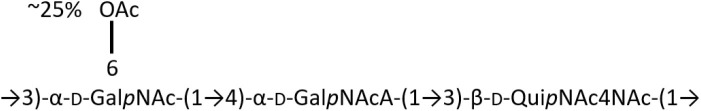	ItrA1	(Kenyon et al., 2016a)
K20	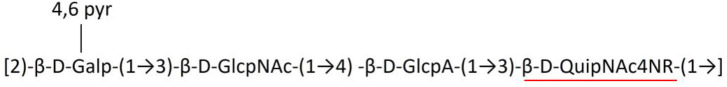	ItrA1	(Kasimova et al., 2018)
K21	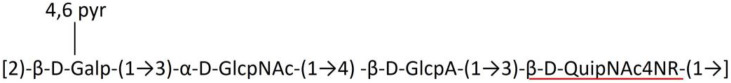	ItrA1	(Kasimova et al., 2018)
K22	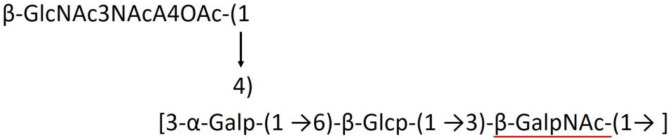	ItrA2	(Talyansky et al., 2021)
K24	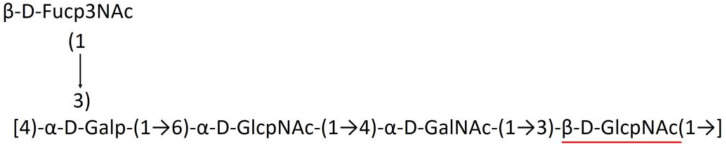	ItrA3	(Kenyon et al., 2017b)
K25	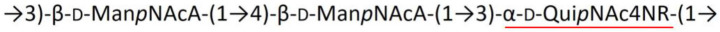	ItrA1	(Senchenkova et al., 2015)
K26	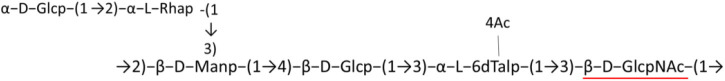	ItrA3	(Kasimova et al., 2021)
K27	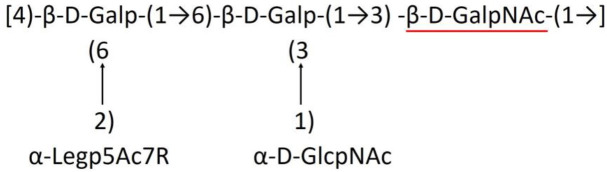	ItrA2	(Shashkov et al., 2016b)
K30	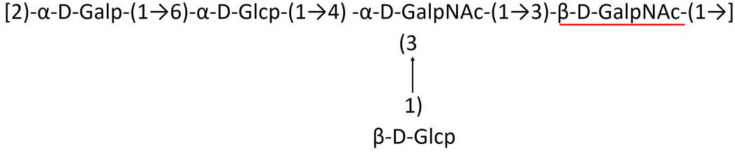	ItrA2	(Shashkov et al., 2015a)
K32	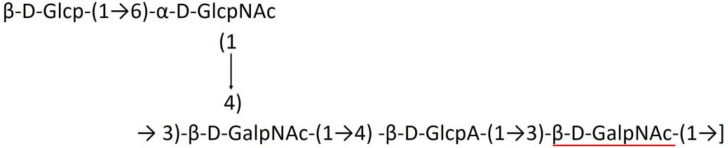	ItrA2	(Cahill et al., 2020)
K33	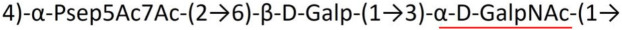	ItrA2	(Arbatsky et al., 2016)
K35	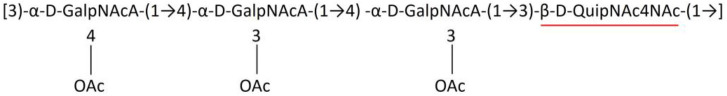	ItrA1	(Shashkov et al., 2017)
K37	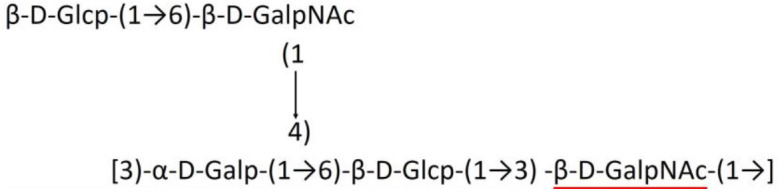	ItrA2	(Shashkov et al., 2019)
K39	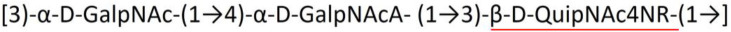	ItrA1	(Kenyon et al., 2016a)
K42	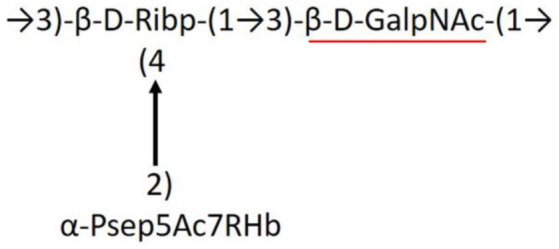	ItrA2	(Senchenkova et al., 2015)
K43	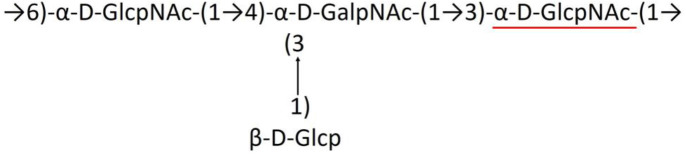	ItrA3	(Shashkov et al., 2016a)
K44	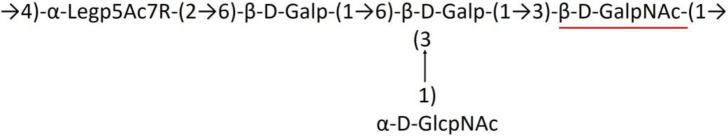	ItrA2	(Shashkov et al., 2016b)
K45	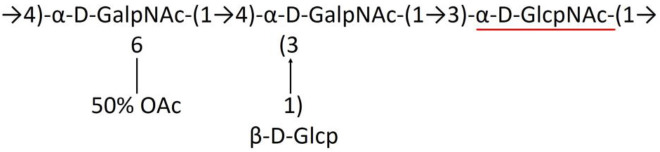	ItrA3	(Shashkov et al., 2015a)
K46	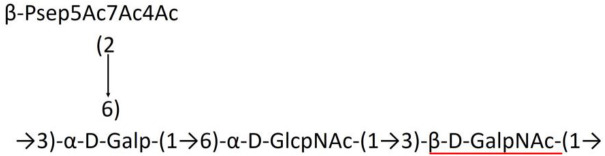	ItrA2	(Kenyon et al., 2019)
K47	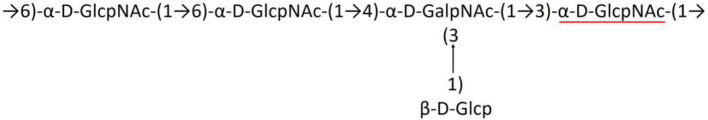	ItrA3	(Shashkov et al., 2016a)
K48	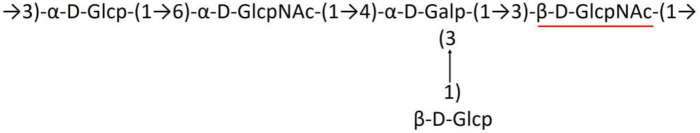	ItrA3	(Shashkov et al., 2015a)
K49	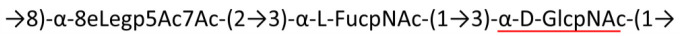	ItrA3; ItrB2	(Singh et al., 2018)
K53	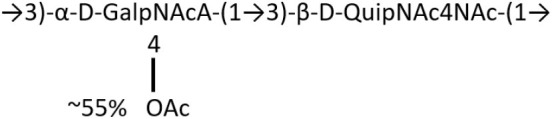	ItrA1	(Shashkov et al., 2018)
K54	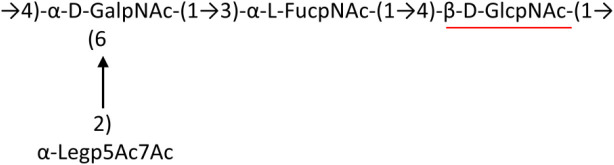	ItrA3; ItrB2	(Arbatsky et al., 2019)
K55	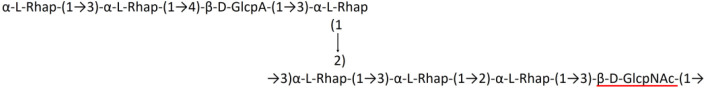	ItrA3	(Kenyon et al., 2021a)
K57	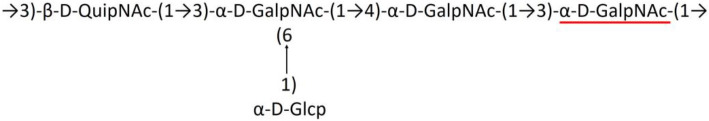	ItrA2	(Kenyon et al., 2018)
K73	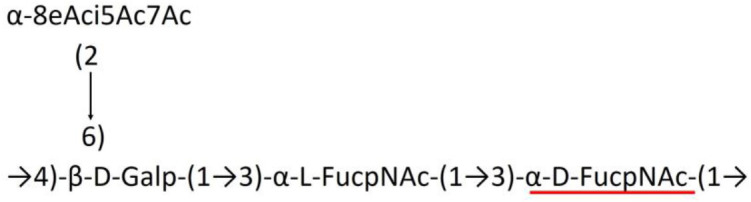	ItrB3	(Kenyon et al., 2017a)
K74	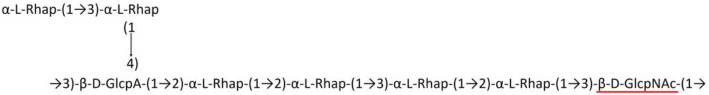	ItrA3	(Kenyon et al., 2021a)
K82	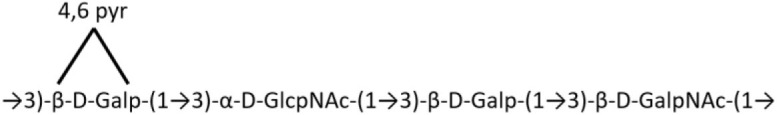	ItrA2	(Kasimova et al., 2018)
K83	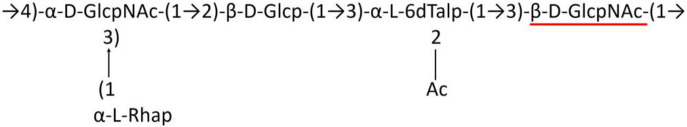	ItrA3	(Kenyon et al., 2017c)
K85	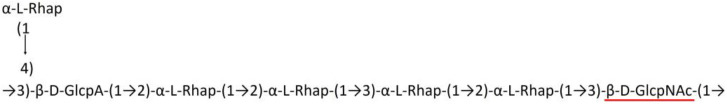	ItrA3	(Kenyon et al., 2021a)
K86	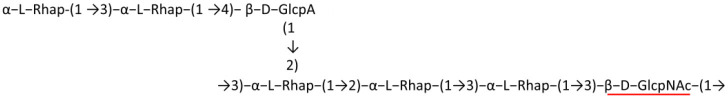	ItrA3	(Kenyon et al., 2021b)
K87	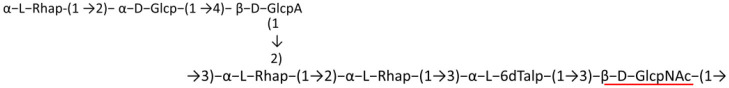	ItrA3	(Arbatsky et al., 2021)
K88	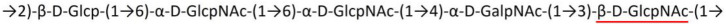	ItrA3	(Shashkov et al., 2016a)
K89	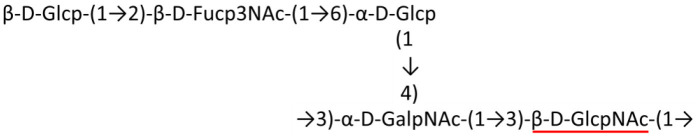	ItrA3	(Arbatsky et al., 2022)
K90	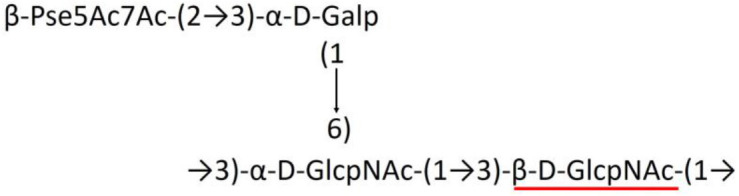	ItrA3	(Senchenkova et al., 2019)
K91	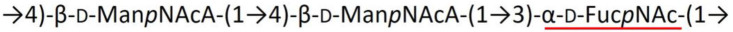	ItrB1	(Shashkov et al., 2015b)
K92	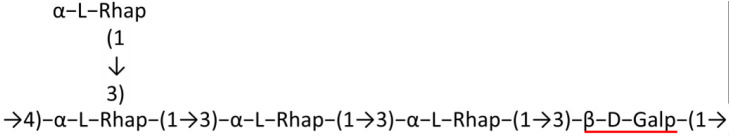	ItrA4	(Senchenkova et al., 2021)
K93	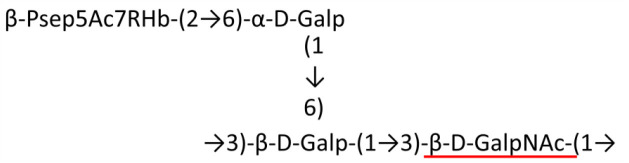	ItrA2	(Kasimova et al., 2017)
K98	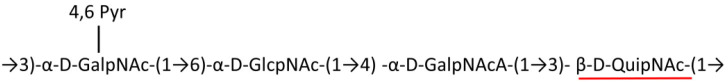	ItrB1	(Kasimova et al., 2022b)
K106	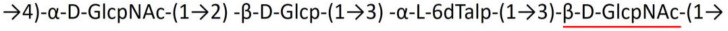	ItrA3	(Kasimova et al., 2021)
K112	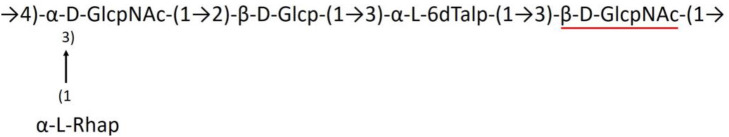	ItrA3	(Kasimova et al., 2021)
K116	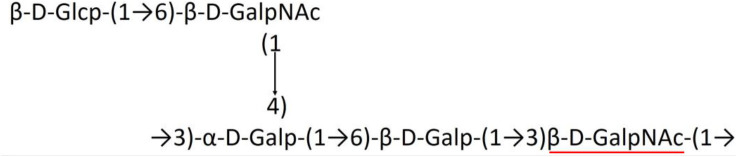	ItrA2	(Shashkov et al., 2019)
K125	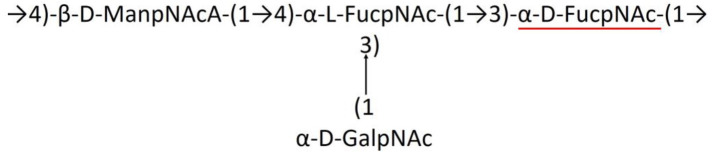	ItrB3	(Arbatsky et al., 2018)
K127	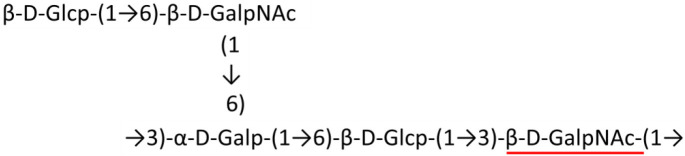	ItrA2	(Arbatsky et al., 2022)
K128	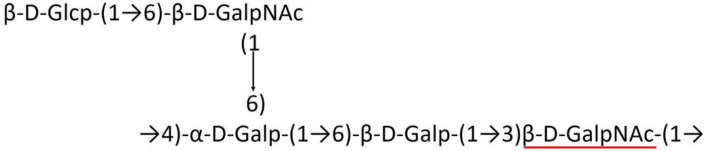	ItrA2	(Arbatsky et al., 2019)
K139	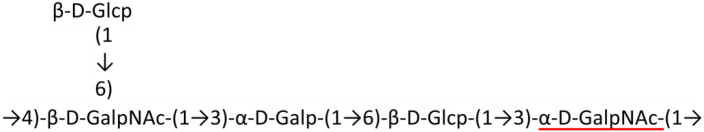	ItrA2	(Kasimova et al., 2021)
K144	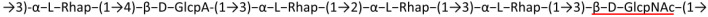	ItrA3	(Kenyon et al., 2021b)
K218	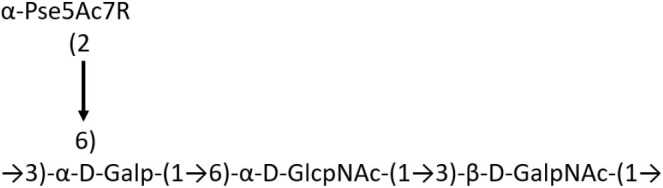	ItrA2	(Kasimova et al., 2022a)

Note that some K-antigens having 2 different sugar substitutions (3-OH-butyrate and acetyl) are also indicated with an R-group.

### Testing the diversity of the proteins for efficient K-typing

The diversity of the 12 protein sequences (notably ItrB4 was not considered as it is present only in KL75) corresponding to 237 serotypes of *A. baumannii* was investigated to effectively utilize them to distinguish different serotypes, as described earlier (Patro et al., 2020). For this, multiple sequence alignment (MSA) and percentage identity matrix (PIM) of all the 12 protein sequences were constructed using Clustal Omega (Madeira et al., 2022). For the multiple sequence alignment of Wza, Wzb, Wzc, Wzx, Wzy, ItrA1, ItrA2, ItrA3, ItrA4, ItrB1, ItrB2, and ItrB3, 259, 244, 256, 246, 248, 35, 91, 82, 5, 13, 20, and 10 sequences were used, respectively. It is noteworthy that the number of Itr sequences is less compared to the other proteins as they are specific only for certain serotypes. Indeed, there was only one sequence available for ItrB4 as it is found only in KL75. Subsequently, the MSA of all these proteins was individually given as input to Weblogo3 for the estimation of each protein's regional diversity by generating their sequence logo (Crooks et al., 2004).

### Calculating the reliability scores

The reliability score (RS) and average reliability score (ARS) for each protein were calculated individually for all 237 serotypes based on the protein's ability to predict a unique serotype when searched against the reference dataset, as described previously (Patro et al., 2020). The reliability score used for the K-typing provides the uniqueness of proteins in the Wzx/Wzy biosynthesis pathway across different serotypes. This has been done by comparing each of the Wzx/Wzy biosynthesis pathway proteins' sequences of different *A. baumannii* K-types using the pairwise sequence alignment method in BLAST (Altschul et al., 1990; McGinnis and Madden, 2004). Notably, the statistical significance value (P) of < 0.01 of BLAST is considered a cutoff for the alignment. See the section “Quantifying the reliability of the Wzx/Wzy-dependent pathway marker proteins in K-typing” under “Results and Discussion” for an explanation of RS with examples. Finally, the average reliability score (ARS) of each protein was calculated individually by averaging the RS values of a protein obtained for all the K-types.

### Implementation of *Acinetobacter baumannii* K-antigen 3-dimensional structure database (ABSD)

The implementation was carried out with the help of the Apache HTTP server (https://httpd.apache.org) and D3.js (https://d3js.org/). While the client-side user interface was implemented using HTML, PHP was used for scripting purposes and to retrieve the K-antigen structures.

## Results and discussion

**Figure 1:**
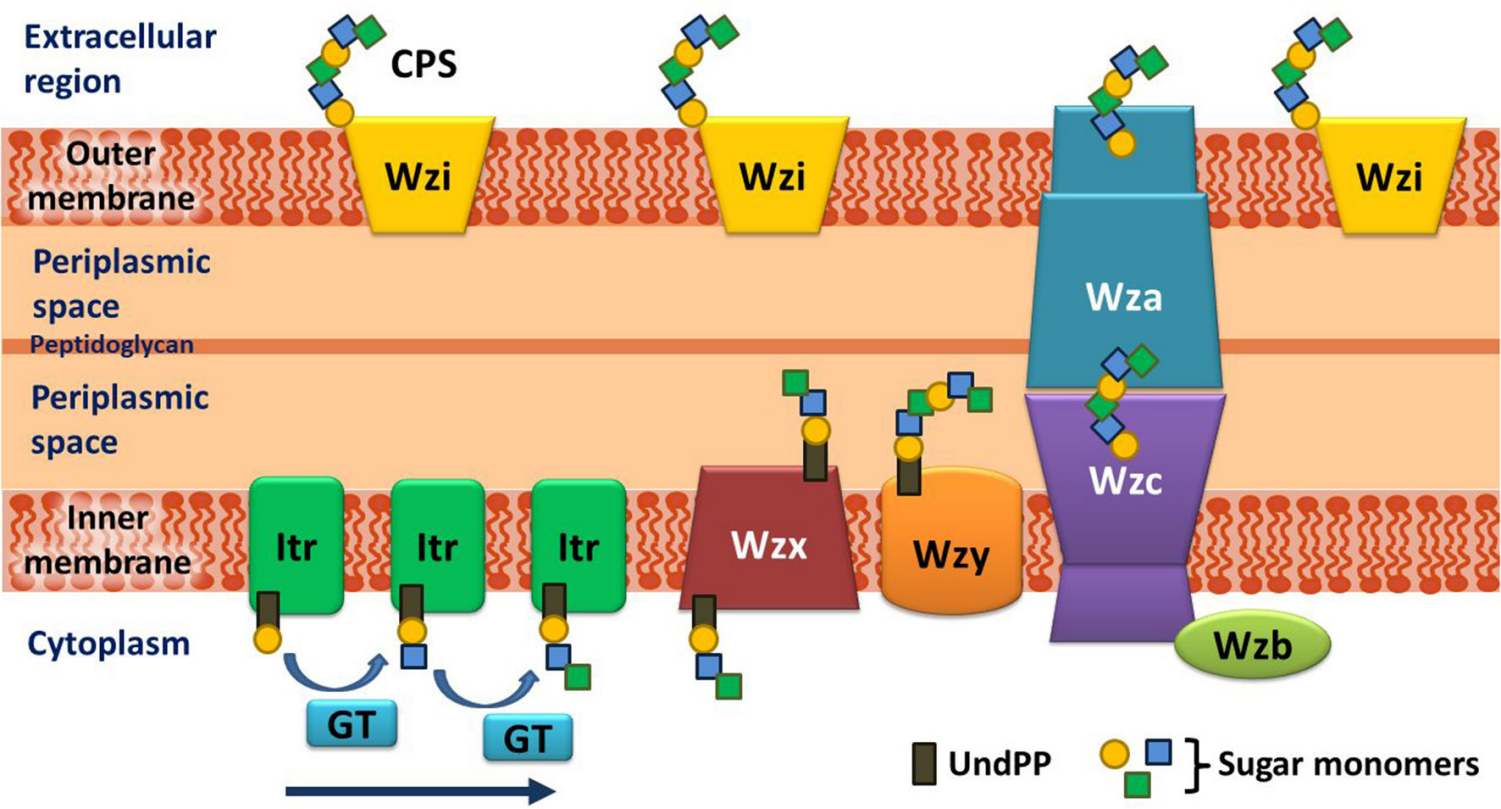
Schematic representation of the assembly and export of capsular polysaccharide (CPS) in *A. baumannii*. The assembly of the repeating unit begins with the transfer of the lipid carrier attached to the first sugar (Und-PP) to the initializing transferase (Itr) located in the inner membrane. Subsequently, the other sugar monomers of the repeating unit are added (indicated by an arrow) by K-type specific glycosyltransferases (GTs) on the cytosolic side of the inner membrane. The CPS repeating unit is, then, transported to the periplasmic region through the Wzx flippase that is located on the inner membrane. The CPS repeating unit is polymerized by the Wzy protein and exported to the cell surface synergistically by Wzc (tyrosine autokinase), Wzb (phosphatase), and Wza (translocon). It is noteworthy that the Wzi protein, which plays a crucial role in retaining the integrity of the CPS layer on the exterior of the outer membrane, is present in a different locus and not in the cps locus of *A. baumannii* (doi.org/10.1038/s41598-021-01206-5), unlike in *Klebsiella* (doi.org/10.3389/fcimb.2019.00367) and *E. coli* (doi.org/10.3389/fmicb.2017.00070).

Before going into the details of the K-antigen structures of *A. baumannii*, an overview of the K-antigen surface exportation machinery is briefed here. *A. baumannii* K-antigen surface assembly and exportation take place *via* the Wzx/Wzy-dependent pathway. The K-antigen repeating unit consists of four to six sugar units unique to each serotype, which further acts as a scaffold for the multimerization to form a mature K-antigen. Several proteins are involved in the K-antigen assembly, multimerization, and surface exportation (Figure 1). The assembly of the K-antigen repeating unit is initialized by initiating transferases (Itrs) in the inner bacterial membrane, followed by the addition of sugar units with the help of glycosyltransferases (Gtrs). This is then followed by the translocation of assembled K-antigen repeating unit into the periplasmic side with the help of Wzx (translocase), and it's polymerization is governed by Wzy (polymerase). Finally, the transport of the growing K-antigen onto the outer membrane takes place through the Wzb–Wzc–Wza complex (Kinsella et al., 2015).

### Classification of *A. baumannii* K-antigens based on the initializing transferases

**Figure 2:**
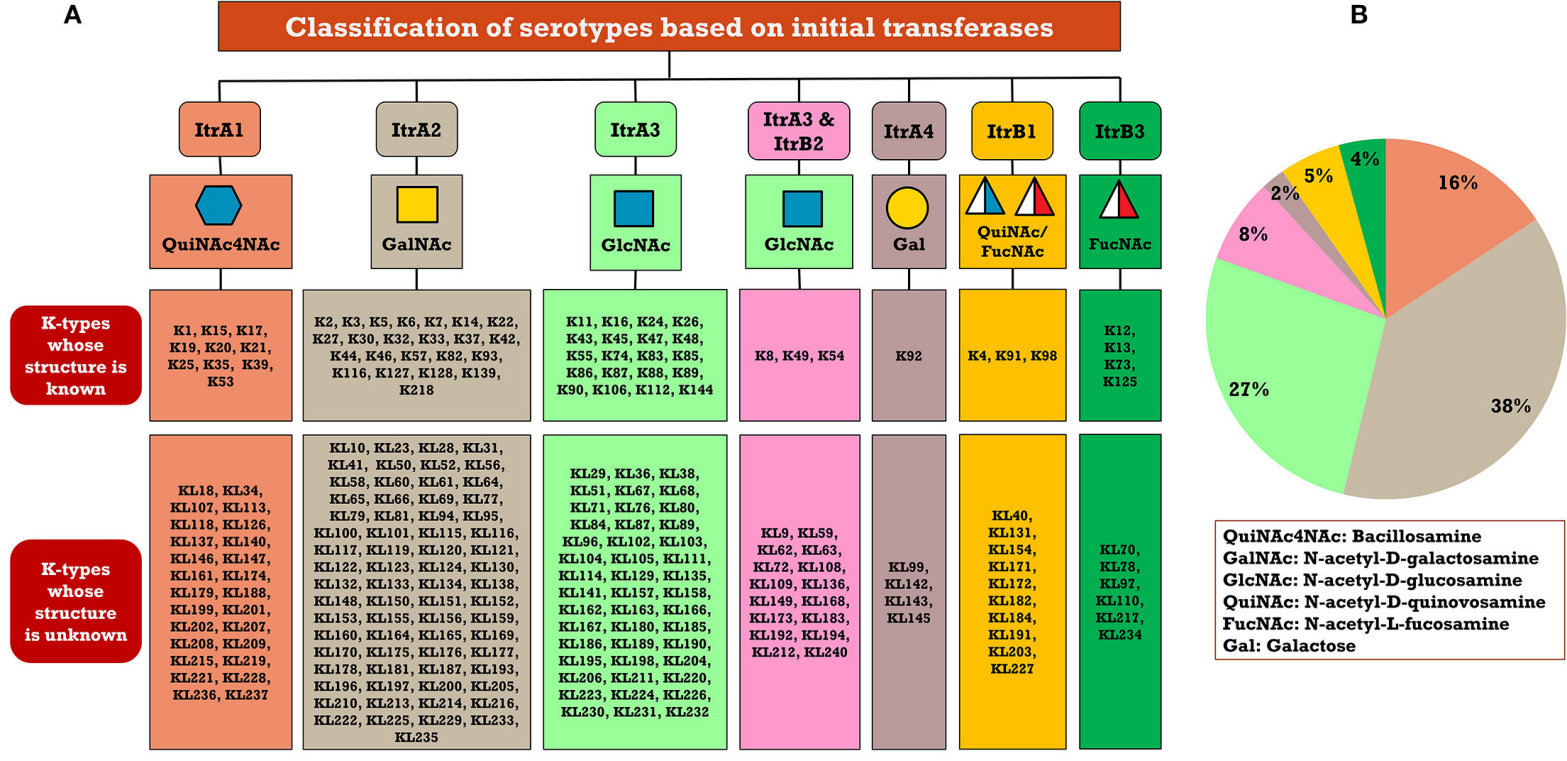
**(A)** Itr-based classification of 237 *A. baumannii* KL-/K-types. **(B)** Pie chart showing the statistics of each initializing transferase. Note that ItrB4 (KL75) and ItrB2 along with ItrA2 (KL115 and KL222) are not marked in **(B)** due to the less frequency of *cps* loci possessing them. Note that Leg5Ac7Ac (or its derivatives) are observed in the K-antigen repeating units that are grouped under the initiating glycosyl transferase ItrA3 & ItrB2.

A critical look at the 237 *A. baumannii* K-antigen gene clusters (Cahill et al., 2022) indicates the presence of eight initializing transferases ItrA1, ItrA2, ItrA3, ItrA4, ItrB1, ItrB2, ItrB3, and ItrB4 that are mutually exclusive except ItrA3 and ItrB2 which occur together. ItrA1, ItrA2, ItrA3, ItrA4, ItrB1, and ItrB3 are associated with QuiNAc4NAc, GalNAc, GlcNAc, Gal, FucNAc/QuiNAc, and FucNAc, respectively (Figure 2A). Interestingly, ItrA3 and ItrB2 are found to occur together, which can be attributed to the presence of GlcNAc as well as Leg5Ac7Ac (or its derivatives), while the individual occurrence of ItrA3 is associated with GlcNAc alone. While ItrB3 is fully responsible for FucNAc, ItrB1 is associated with FucNAc or QuiNAc. A deeper look at the *A. baumannii* K-antigen gene clusters (Cahill et al., 2022) indicates that it can be classified into seven groups based on the aforementioned initializing transferases (Itrs), similar to that in *Salmonella* spp. (Sundaresan and Rathinavelan, 2023) and *Klebsiella* spp. (Patro and Rathinavelan, 2019). Since the presence or absence of an Itr depends on the first sugar of the K-antigen, these are mutually exclusive in general. Thus, the initial sugar would act as a valuable index for the classification of K-antigens. Surprisingly, only one *cps* locus (KL75) is found to have a rare initializing transferase, ItrB4, which is ~81% identical to ItrB3 sequences (https://project.iith.ac.in/ABSD/data_abs/pim-all-Itrs-PP.txt). Furthermore, two *cps* loci (KL115 and KL222) are found to have ItrB2 along with ItrA2. Figure 2 shows the classification of K-antigens based on different Itrs (*viz*., initial sugars). Notably, GalNAc is the most preferred initial sugar with a relative percentage of 38% (Figure 2B), followed by GlcNAc (27%), QuiNAc4NAc (16%), GlcNAc and Leg5Ac7Ac (or Leg5Ac7Ac derivatives) (8%), QuiNac/FucNAc (5%), FucNAc (4%), and Gal (2%). Among the 237 K-antigens of *A. baumannii*, only 64 K-antigen structures are known. Table 1 presents the published chemical representations of the 64 K-antigens of *A. baumannii*, wherein the sugar monomers of the repeating unit and their glycosidic linkages are derived using NMR (Russo et al., 2013; Arbatsky et al., 2022) and Smith degradation (Arbatsky et al., 2022). Exceptionally, the K19 structure was derived from the structure of K39 through the addition of an acetyl group to K39 (Kenyon et al., 2016a).

A detailed analysis of the sugar composition indicates that there are 25 unique sugars found in the K-antigen main chain apart from the initial sugars, and 19 unique sugars are seen in the side chain (Table 2). Notably, *A. baumannii* K-antigen structures are found to have derivatives of common sugars. For instance, pseudaminic acid, legionaminic acid, and bacillosamine are the derivatives of mannose, neuraminic acid, and glucose, respectively. Apart from this, many substitutions are also seen, among which, N-acetylation and O-acetylation of the sugar ring are predominant. This is followed by acetylation (of the exocyclic atoms of the sugar), pyruvic acid, and D-alanine substitutions. Realizing the importance of the charge of K-antigens (alternatively, CPS) in supporting the bacteria to escape from the host immune response (*viz*., phagocytosis (Moxon and Kroll, 1990)) and providing resistance to antimicrobial peptides (Band and Weiss, 2015) and antibiotics (Aska Fang, 2017), the charge of each K-antigen is explored here. Interestingly, 32.8% of the K-antigens of *A. baumannii* are neutrally charged and others are negatively charged (Table 2). Among the negatively charged K-antigens, 53.1%, 12.5%, and 1.6% have a charge of −1, −2, and −3, respectively. While the negative charge in K1, K3, K4, K15, K17, K19, K20, K21, K22, K25, K32, K35, K37, K39, K53, K55, K74, K85, K86, K87, K91, K98, K125, and K144 is due to the presence of a carboxyl group at the C6 position, the negative charge of K4, K20, K21, K82, and K98 is provided by the pyruvic acid.

**Table 2:** The main- and side-chain sugar compositions of *A. baumannii* K-antigens are presented in Table 1.

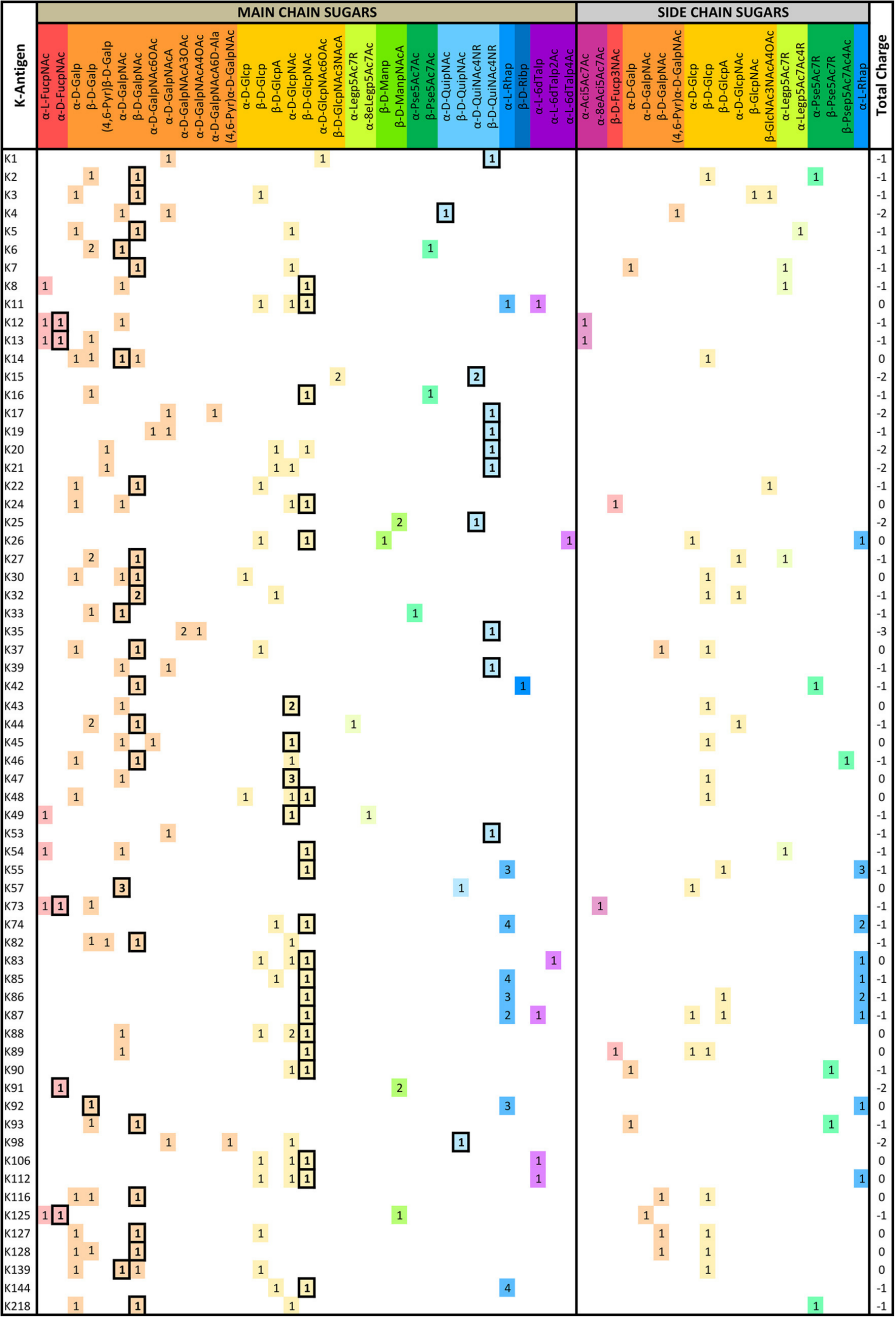

The initial sugar is indicated by a box. Note that the total charge of each K-antigen is also indicated in the last column.

### Creation of *A. baumannii* K-antigen 3D-structural repository

**Figure 3:**
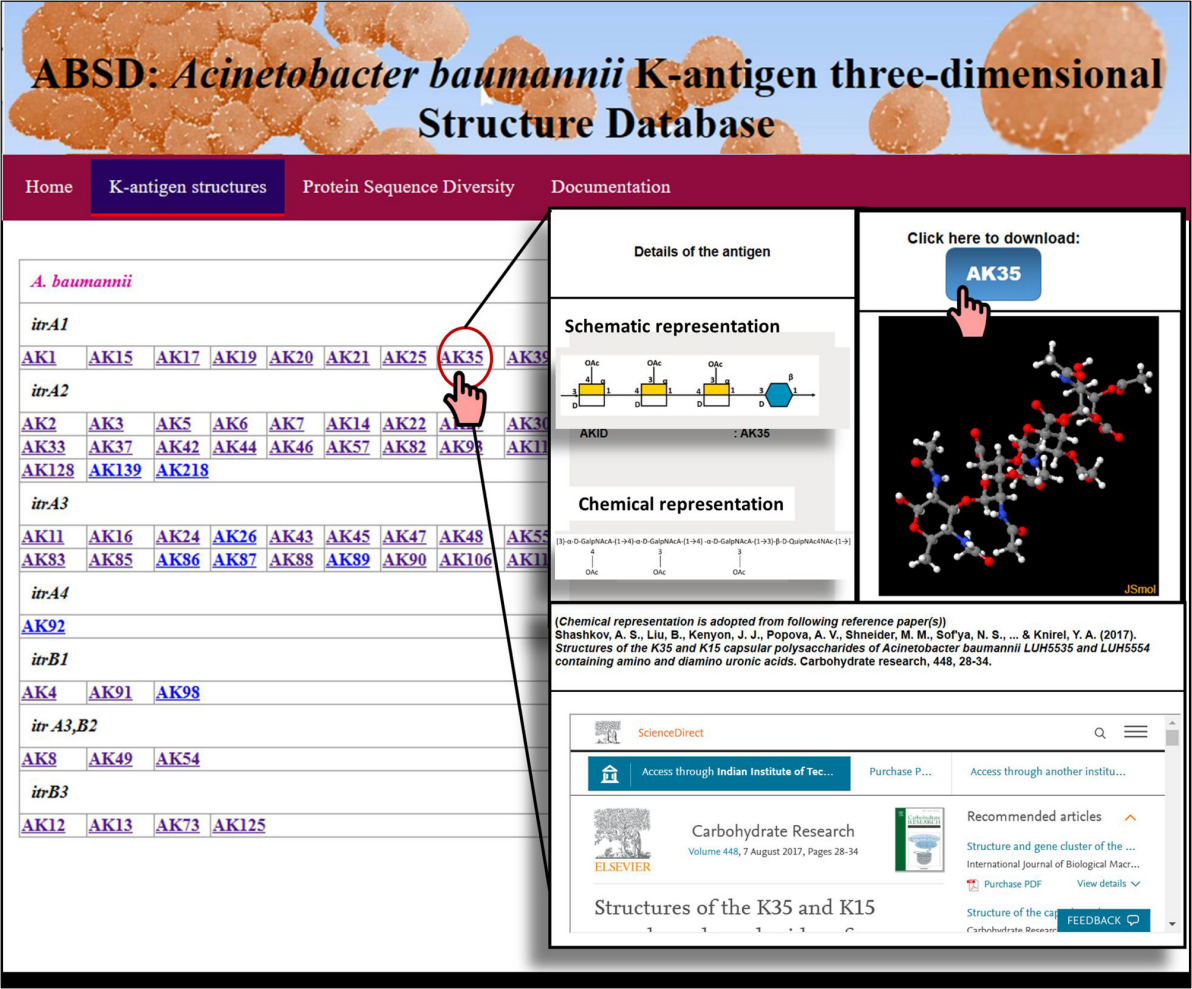
A snapshot of the *A. baumannii* K-antigen structure repository, ABSD.

The structural information presented in Table 1 was utilized to model and create the 3D structural repository of *A. baumannii* K-antigen, similar to the K- and O-antigen structural repositories of *E. coli* (Rojas-Macias et al., 2015; Kunduru et al., 2016) and *Klebsiella* spp. (Patro et al., 2020). Here, 64 known K-antigen three-dimensional structures were modeled, and their repository was created in a Linux-based server (Figure 3). For the K-antigens having more than one substitution (refer to Table 1), the model with one of the substitutions was generated using the protocol discussed in the Materials and Methods section and was used as a template to model the second substitution using Pymol (Schrodinger, 2015). The K-antigens can be accessed through the K-antigen structure module of *Acinetobacter baumannii* K-antigen three-dimensional Structure Database (ABSD): https://project.iith.ac.in/ABSD/k_antigen.html. The module permits the user to either visualize the K-antigens interactively using the JSmol viewer (Robert et al., 2013) or download the coordinates. The structure of the appropriate K-antigen can be accessed by clicking the antigen ID, as shown in Figure 3.

### Main chain and side chain topology of *A. baumannii* K-antigens

**Figure 4:**
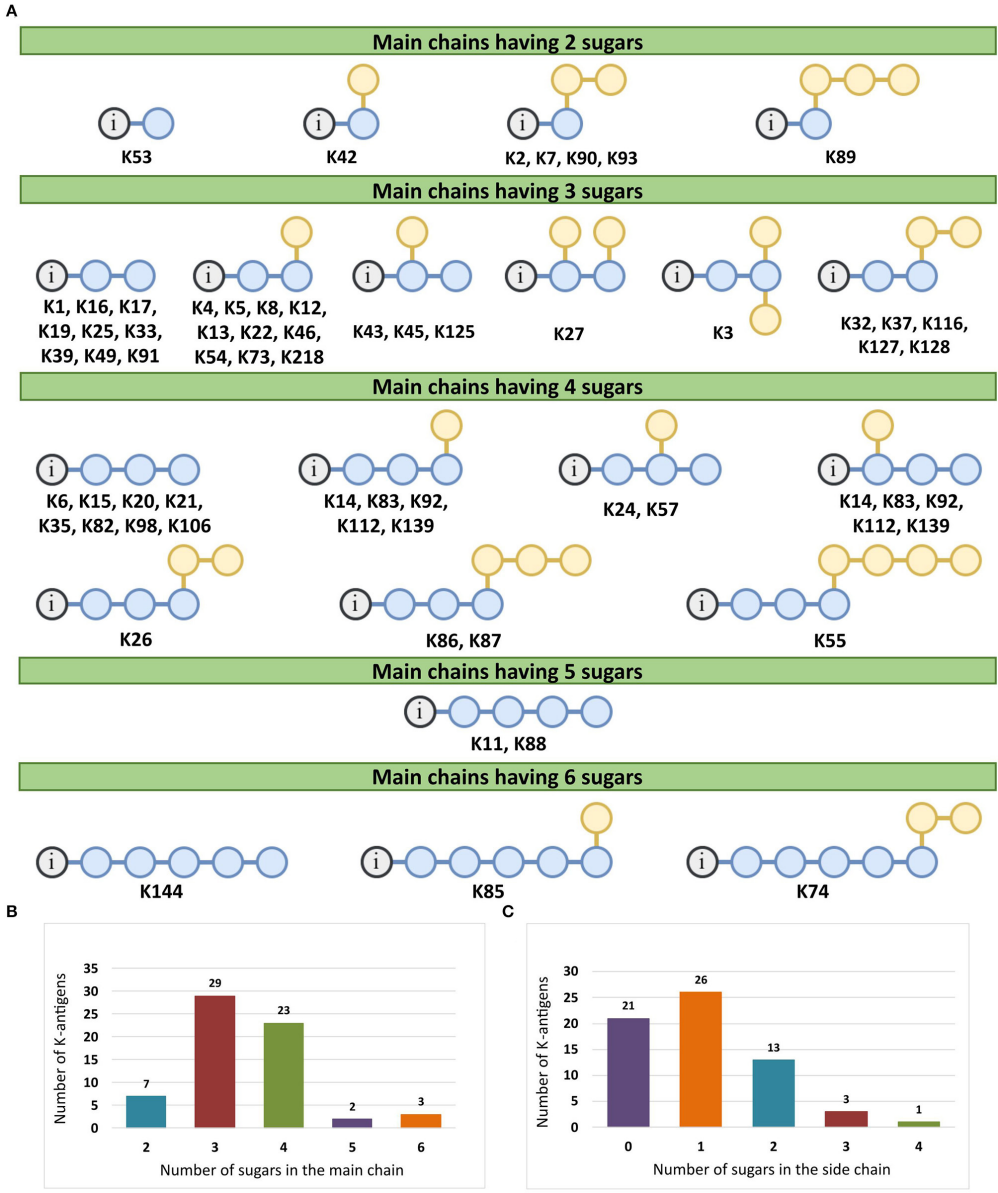
**(A)** Schematic representation of the main (blue circles) and side (yellow circles) chain topologies of the 64 K-antigens of *A. baumannii* whose chemical structures are known (Table 1). Note that ‘i' represents the initial sugar of the respective K-antigen. Bar chart showing the number of sugar monomers in the 64 K-antigens **(B)** main and **(C)** side chains.

There are 21 unique topologies (Figure 4A) for the 64 *A. baumannii* K-antigens whose chemical formulae (Table 1) are known. In the main chain, the sugar monomers are found to be in the range of 2 to 6. There are 7, 29, 23, 2, and 3 K-antigens having 2, 3, 4, 5, and 6 sugar monomers in their main chain, respectively (Figure 4B), among which, the K-antigens having 3 and 4 monomers in the backbone are quite dominant. Most of the repeating units have only one branch except for K3 and K27, which have two branches (Table 1). Some K-antigens have 2 (K53), 3 (K1, K16, K17, K19, K25, K33, K39, K49, and K91), 4 (K6, K15, K20, K21, K35, K82, K98, and K106), 5 (K11 and K88), and 6 (K144) sugars in the backbone but do not have any branches (Figure 4A). The K-antigens with branches have a maximum of four sugar units (Figure 4C). The majority of the branches have only one sugar.

### Sequence diversity analysis of the 12 proteins involved in the Wzx/Wzy-dependent pathway

The divergence in the sugar composition and scaffold of 64 known K-antigen structures of *A. baumannii* (Tables 1, 2, Figure 4) suggests that the proteins involved in their assembly and surface transport may be exhibiting divergence as seen in the *Klebsiella* spp. (Patro et al., 2020). Thus, the diversity of the protein sequences involved in the K-antigen assembly and transport (Figure 1) corresponding to all the 237 K-types of *A. baumannii* was investigated to understand their efficacy in distinguishing different serotypes. For this, multiple sequence alignment (MSA) and percentage identity matrix (PIM) (https://project.iith.ac.in/ABSD/PIM.php) of all the 12 protein sequences were constructed using Clustal Omega (Madeira et al., 2022). The sequences were collected from either NCBI GenBank or Kaptive's *A. baumannii* K-locus reference dataset (Wyres et al., 2020; Cahill et al., 2022) (Supplementary Table S2). PIM indicates that among all the Itrs (https://project.iith.ac.in/ABSD/PIM.php, Supplementary Table S3), ItrA3 is quite diverse between different K-types [average percentage identity (API)=87.95% (standard deviation = 6.27)], whereas the other Itrs share a good sequence identity among different K-types, resulting in an average percentage identity >94%. Among different Itrs, ItrB1, ItrB3, and ItrB4 share a good sequence identity, ItrB1 and ItrB3 have an API of 70.3%, ItrB3 and ItrB4 have an API of 81.9%, and ItrB1 and ItrB4 have an API of 71.3%. Following these, ItrA1, ItrA2, and ItrA3 share a good sequence identity among themselves (API in the range of 60.3% to 74.4%). The polymerase Wzy exhibits the lowest PIM among different K-types, which is proceeded by Wzx, Wza, Wzb, and Wzc. The regions of diversity of all these protein sequences were subsequently determined by generating sequence logo results (Supplementary Figures S1–S12) obtained from Weblogo3 (Crooks et al., 2004). The sequence logos corresponding to 12 proteins involved in the Wzx/Wzy-dependent pathway indicate that while ItrA1 (Supplementary Figure S1), ItrA2 (Supplementary Figure S2), and ItrA4 (Supplementary Figure S4) are highly conserved, ItrA3 sequences are divergent at the N- and C-termini (Supplementary Figure S3). While ItrB1 is a little divergent at the N-terminal end (Supplementary Figure S5), ItrB2 (Supplementary Figure S6) and ItrB3 (Supplementary Figure S7) are highly conserved. Compared to the Itrs, Wza (Supplementary Figure S8), Wzb (Supplementary Figure S9), and Wzc (Supplementary Figure S10) exhibit a bit more divergence. However, Wzx (Supplementary Figure S11) and Wzy (Supplementary Figure S12) sequence logos show that they are the most divergent among different KL-serotypes of *A. baumannii*; thus, they can be more reliable in predicting the K-serotype. Due to their mutually exclusive nature and lesser sequence identity (below 75%), the Itrs can readily be used to distinguish different K-antigen groups, as shown in Figure 2. Notably, Wzi, which anchors the K-antigen on the outer membrane, is excluded as it lies outside the CPS locus. Indeed, it is highly conserved among different K-types (Tickner et al., 2021), and thus cannot be used to distinguish different K-types.

### Quantifying the reliability of the Wzx/Wzy-dependent pathway marker proteins in K-typing

Since traditional serotyping techniques such as agglutination (Traub, 1989), Smith degradation (Arbatsky et al., 2022), one- and two-dimensional NMR (Russo et al., 2013; Arbatsky et al., 2022), and mass spectrometry (Russo et al., 2013) are time-consuming and laborious, *in silico* serotyping has been shown to be effective in Gram-negative bacteria (Wick et al., 2018; Patro et al., 2020; Sundaresan and Rathinavelan, 2023). It has been shown earlier for *Klebsiella* spp. that the reliability score (RS) of the individual Wzx/Wzy-dependent pathway proteins provides quantitative information about the degree of uniqueness of these proteins to the respective KL types (or K-types) compared to the PIM. Thus, the individual RS (Supplementary Table S4) values for the 13 protein sequences (Wzc, Wzy, Wza, Wzx, Wzb, ItrA4, ItrA3, ItrA2, ItrA1, ItrB4, ItrB3, ItrB2, and ItrB1) were estimated across 237 K-types of *A. baumannii*, as described elsewhere (Patro et al., 2020), with the help of an automated bash script that searches a protein sequence of a K-antigen (for example, Wzc of K3) against the same protein sequences of the remaining K-antigens. In brief, when a protein sequence was identical to two different serotypes, the RS was calculated to be 50%. Nonetheless, the RS was calculated to be 100% if a protein sequence (for example, Wzc of K45) was unique to a particular serotype. Finally, an average reliability score (ARS) (last row of Supplementary Table S4) of each protein was individually calculated by averaging the RS values of a protein across all the K-types. Notably, BLAST (Altschul et al., 1990; McGinnis and Madden, 2004) was employed to compare two protein sequences, for which a sequence identity cutoff of 60% was used. ARS was estimated to get information about the highly reliable protein(s) among the 13 CPS proteins for the accurate K-type prediction of *A. baumannii*. The ARS, which is calculated using the RS of all the sequences belonging to 237 K-types, falls in the following order:

Wzy>Wzx>ItrB3>Wzc>ItrA4>ItrA1>Wzb>ItrA3>Wza >ItrB1>ItrB2>ItrA2

Notably, ItrB4 is 100% reliable as it is found only in one K-type (KL75).

As shown above, the ARS value indicates that the degree of reliability is the highest for Wzy and the lowest for ItrA2. Thus, Wzy can be efficiently used in the K-serotyping of *A. baumannii*. However, the ARS is only 69.25% including for Wzy, which indicates that the use of Wzy alone may lead to multiplicity in K-typing. Thus, as shown earlier in *Klebsiella* spp., serotyping involving multiple proteins in the K-type prediction may reduce the K-type prediction multiplicity (Patro et al., 2020). Overall, these analyses give a clue that the 13 protein sequences of the Wzx/Wzy-dependent pathway can be used together in the accurate K-serotyping of *A. baumannii*, as in the case of *Klebsiella* spp. (Patro et al., 2020). Notably, in the case of *A. baumannii* reference sequences, 182 of the 237 KL-types were correctly identified (without any multiplicity), with the help of Wzx/Wzy-dependent pathway proteins (last column of Supplementary Table S4).

**Figure 5:**
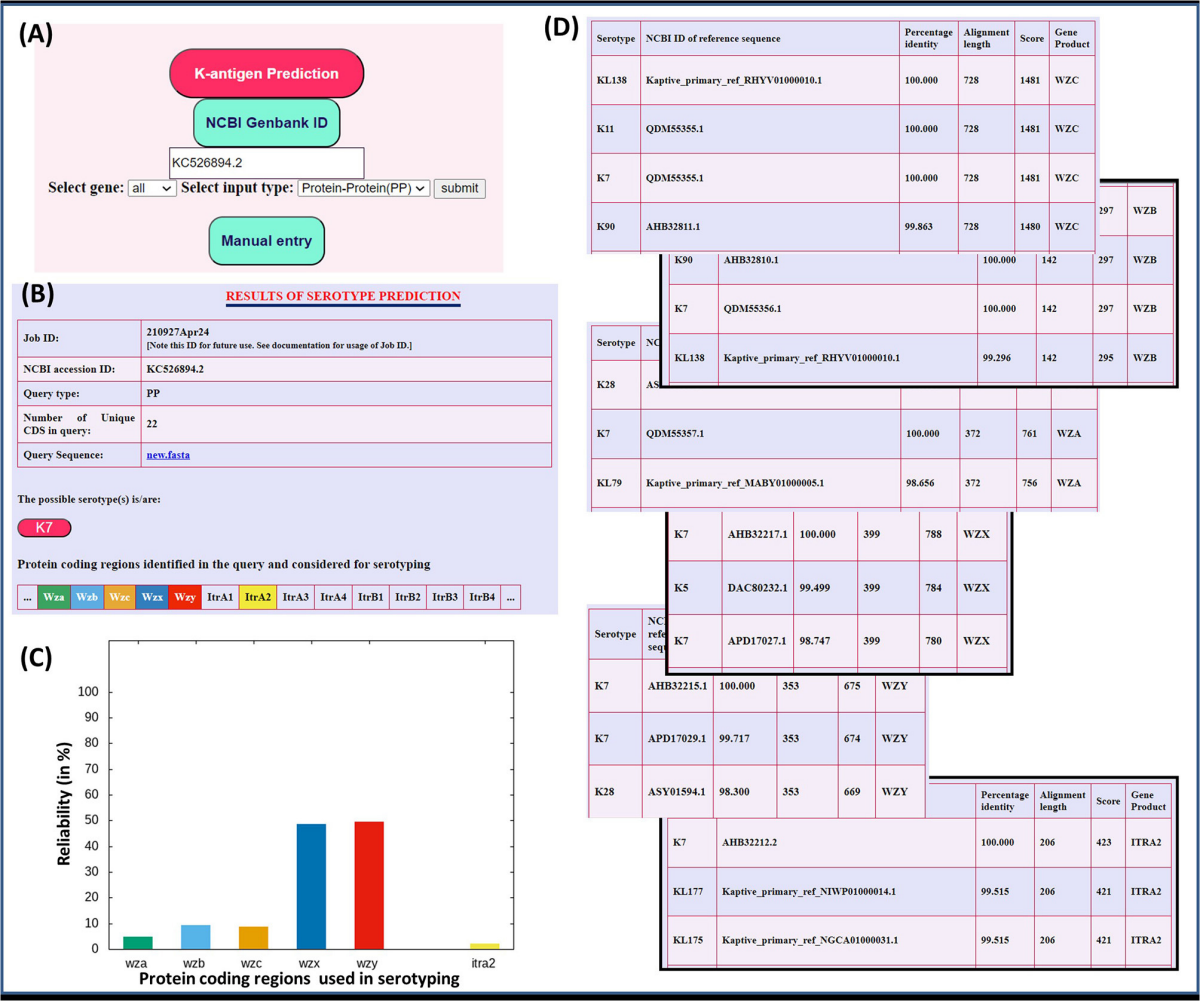
Utilization of initial glycosyl transferases (Itrs) and Wza, Wzb, Wzc, Wzx, and Wzy proteins in the K-type prediction of *A. baumannii*. **(A)** Input page of the serotype predictor module. The input sequence can either be directly provided through its NCBI accession ID (here, NCBI ID: KC526894.2 is taken as an example) or manually pasted in the given box, or uploaded as a FASTA format file. **(B–D)** are the outcomes displayed on the result page: **(B)** The details of the input sequence, predicted serotype, and protein-coding regions considered for serotyping are displayed in the top part of the result page. **(C)** The reliability (in percentage) of the serotype prediction from the alignment of individual protein-coding regions is represented through a graph (see text for details). **(D)** The top hits corresponding to the serotype predictions from the individual proteins are summarized in tables. Notably, the tables show the multiplicity in the serotype prediction using the individual proteins, which is overcome by the use of multiple proteins, thus providing a single serotype.

In short, the presence of initial glycosyl transferases can be used in the first place to identify the K-antigen having the concomitant initial sugar (Figure 2). Subsequently, the other proteins can be used for the serotype prediction based on their percentage identity between different proteins, as discussed above. To elucidate this point, the “Serotype predictor” module is enabled, which uses the initial glycosyl transferases (Itrs) and Wza, Wzb, Wzc, Wzx, and Wzy proteins for the serotype prediction (Figure 5). The methodology is the same as the one described for *Klebsiella* spp. (Patro et al., 2020), thus not described in detail (Supplementary Figure S13). By considering the whole genome sequence corresponding to NCBI ID: KC526894.2 as a test case, an example illustrating the use of these proteins in *in silico* K-typing of *A. baumannii* is given in Figure 5. A few more examples are given in Supplementary Figures S14–S15. In the example shown in Supplementary Figure S14, the Wzy of K13 is not highly specific, it has an RS value of 33%. Thus, the utilization of other proteins along with Wzy improves serotyping accuracy. This is demonstrated by considering NCBI Accession ID: CP050388.1, wherein the combination of the Wzx/Wzy-dependent pathway proteins accurately predicts the K-type (Supplementary Figure S14).

## Conclusion

Here, we have presented 64 K-antigen structures of *A. baumannii* which form 21 different topologies. The K-antigen structures are diverse in terms of sugar composition and their charges (neutral as well as negatively charged). The mutually exclusive nature of the Itrs and their sequence diversity among themselves facilitate the Itr-based classification of *A. baumannii* K-antigens. Similarly, the variations in the K-antigen structures and the concomitant sequence diversity of 13 K-antigen assembly and transport proteins involved in the Wzx/Wzy-dependent pathway indicate their ability in fast and accurate K-typing. Furthermore, the *A. baumannii* K-antigen structural information provided here would not only be useful in the phage therapy and design of vaccines and antibiotics but also would help in understanding the interaction of this pathogen with the host.

## Data availability statement

The datasets presented in this study can be found in online repositories. The names of the repository/repositories and accession number(s) can be found below: https://project.iith.ac.in/ABSD/k_antigen.html.

## Author contributions

JR collected and modeled *A. baumannii* chemical structures and sequences used in the preliminary analysis. LP implemented the web tool and updated the sequence dataset. SS analyzed the K-antigen structure and topology and updated the antigen three-dimensional structure database. SS, TR, and LP wrote the manuscript. TR designed and supervised the project. All authors contributed to the article and approved the submitted version.
