# Supplementary material for: Structural diversity among Acinetobacter baumannii K-antigens and its implication in the in silico serotyping
Source: Front Microbiol. 2023 Jun 21;14:1191542. doi: 10.3389/fmicb.2023.1191542 (PMC10320297; doi:10.3389/fmicb.2023.1191542)
Supplement: Supplementary file 5 [file Data_Sheet_1.DOCX]

**Structural diversity among *Acinetobacter baumannii* K-antigens and its implication in the *in silico* serotyping**

Janardhanaachari Roshini, L Ponoop Prasad Patro^†^, Sruthi Sundaresan^†^ and Thenmalarchelvi Rathinavelan*

Department of Biotechnology, Indian Institute of Technology Hyderabad, Kandi,

Telangana State 502284, India

^†^These authors contribute equally

*For Correspondence: tr@bt.iith.ac.in

**Running title:**

***A. baumannii*** **marker proteins' K-typing specificity**

**Keywords:** *Acinetobacter baumannii*, K-antigen structure, capsular polysaccharide, antimicrobial resistance, K-typing, Wzx/Wzy-dependent pathway

**Supplementary tables and figures**

**Table S1.** A few examples showing the clinical relevance of different K-types (or K-locus (KL) types) of *Acinetobacter baumannii*.

**Table S2.** GenBank accession IDs of the protein sequences involved in the K-antigen assembly and transport (Figure 1) corresponding to all the 237 K-types. Note that some of the sequences were taken directly from Kaptive's Acinetobacter_baumannii_k_locus_primary_reference dataset. (https://github.com/katholt/Kaptive/blob/master/reference_database/Acinetobacter_baumannii_k_locus_primary_reference.gbk).

**Table S3.** Percentage identity matrix built from Clustal-Omega server for all the Itr sequences. Note that the columns are colored according to the Itrs, as shown in Figure 2.

**Table S4.** Reliability scores were calculated for Wzx/Wzy-dependent pathway proteins that correspond to 237 K-types of *A. baumannii*. Note that each protein's average reliability scores are given in the last row. It is to be noted that the values were estimated by considering a sequence identity cut-off of 98% and "NA" indicates that the corresponding sequence was not found in the reference dataset.

| 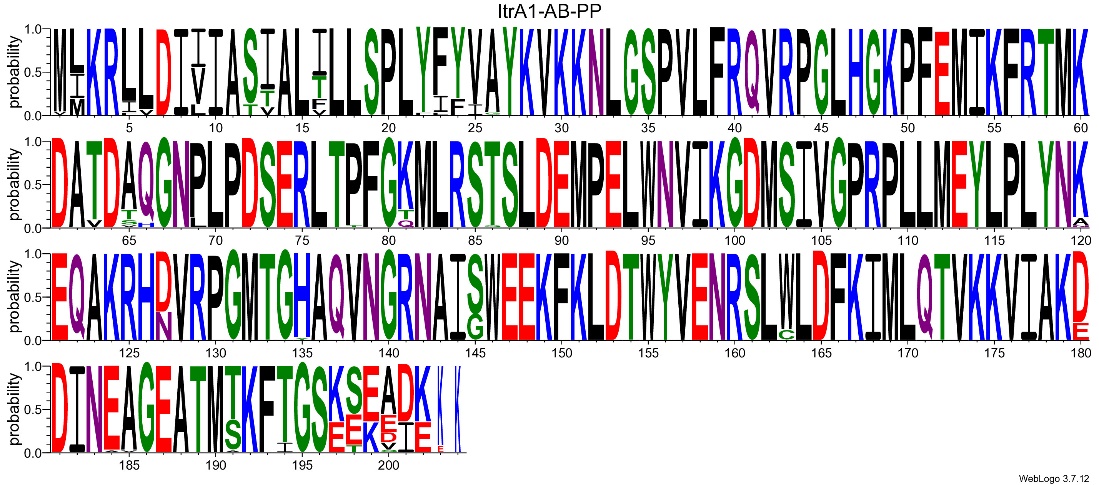 |
| --- |
| **Figure S1.** Weblogo reflecting the sequence diversity/similarity of ItrA1 among the 237 KL-types of *A. baumannii*. |

| **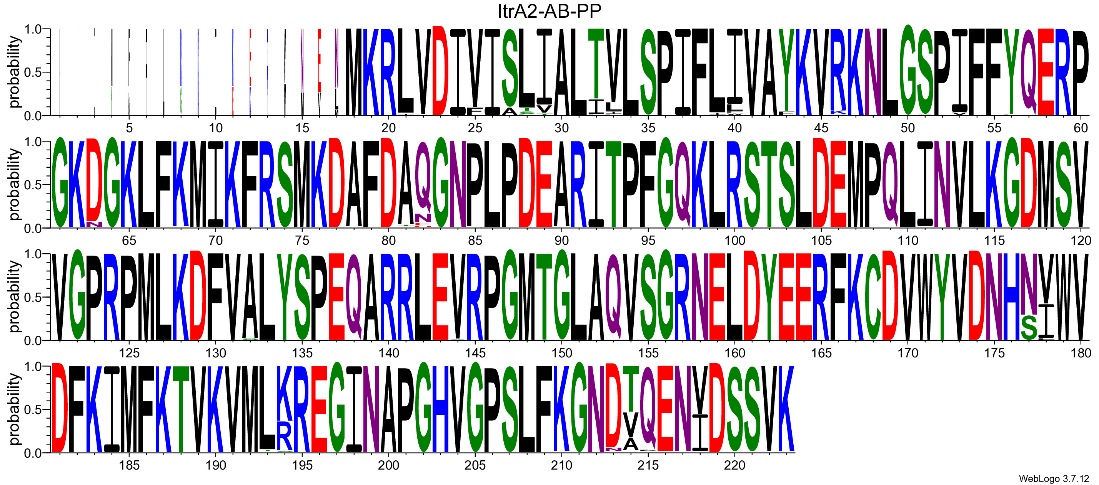** |
| --- |
| **Figure S2.** Weblogo reflecting the sequence diversity/similarity of ItrA2 among the 237 KL-types of *A. baumannii*. |

| **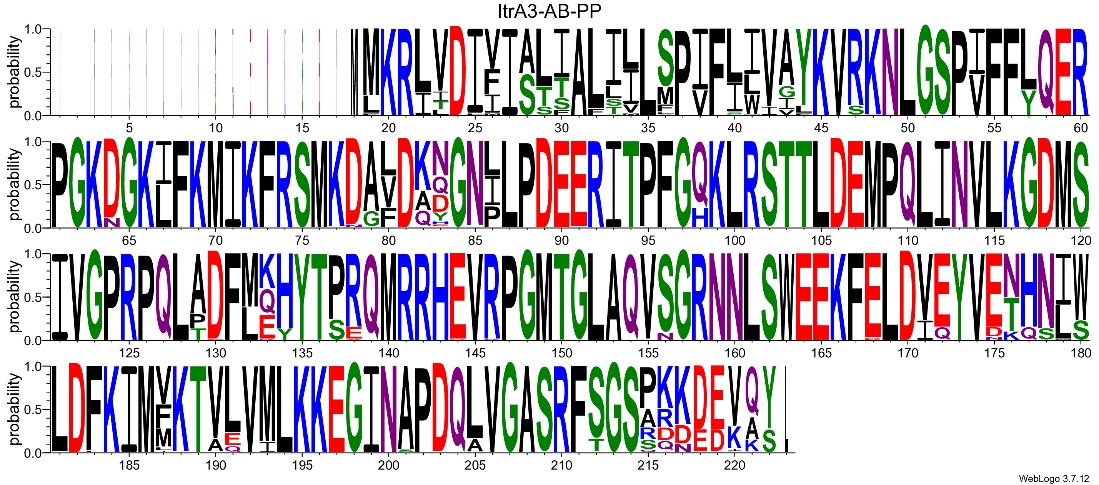** |
| --- |
| **Figure S3.** Weblogo reflecting the sequence diversity/similarity of ItrA3 among the 237 KL-types of *A. baumannii*. |

| **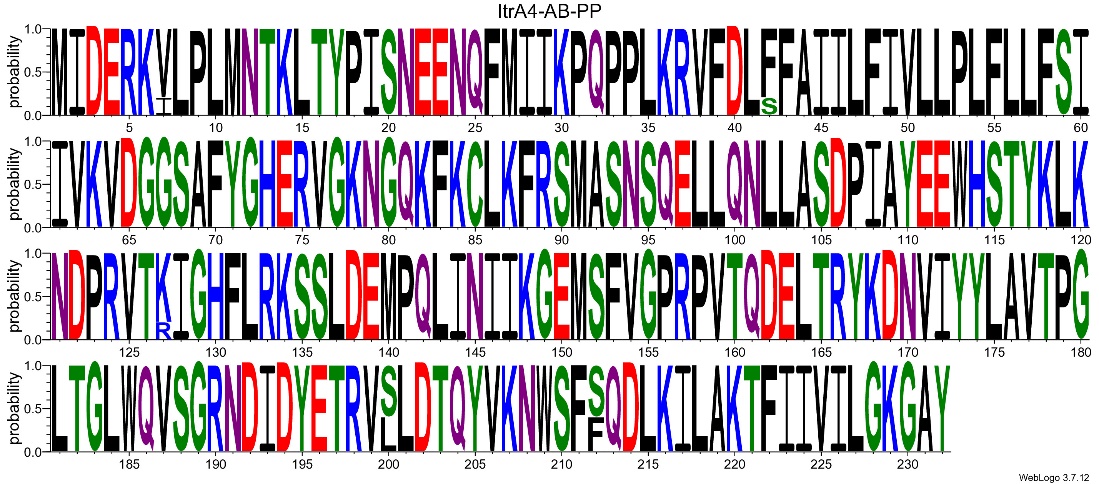** |
| --- |
| **Figure S4.** Weblogo reflecting the sequence diversity/similarity of ItrA4 among the 237 KL-types of *A. baumannii*. |
| **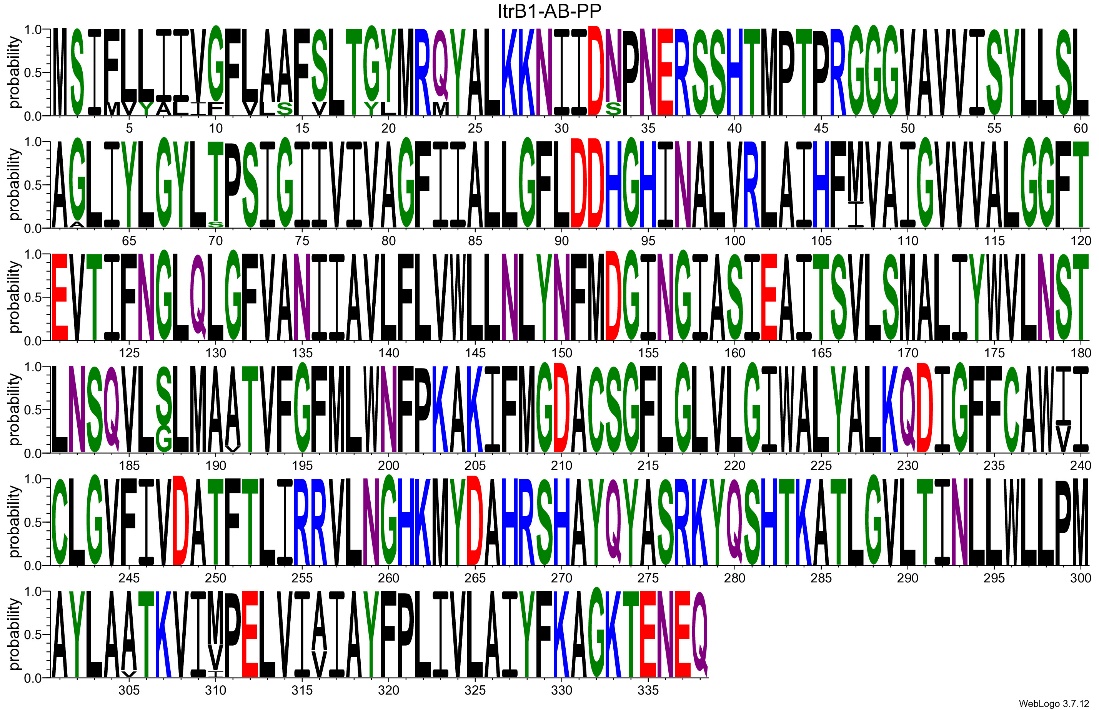** |
| **Figure S5.** Weblogo reflecting the sequence diversity/similarity of ItrB1 among the 237 KL-types of *A. baumannii*. |

| **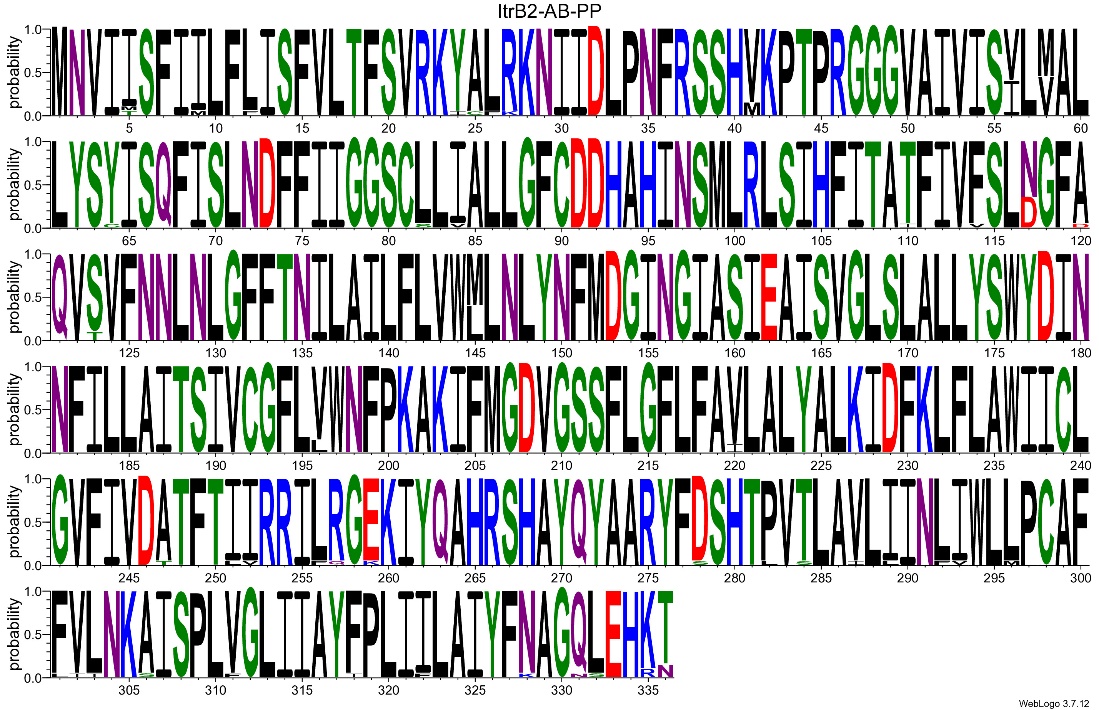** |
| --- |
| **Figure S6.** Weblogo reflecting the sequence diversity/similarity of ItrB2 among the 237 KL-types of *A. baumannii*. |

| **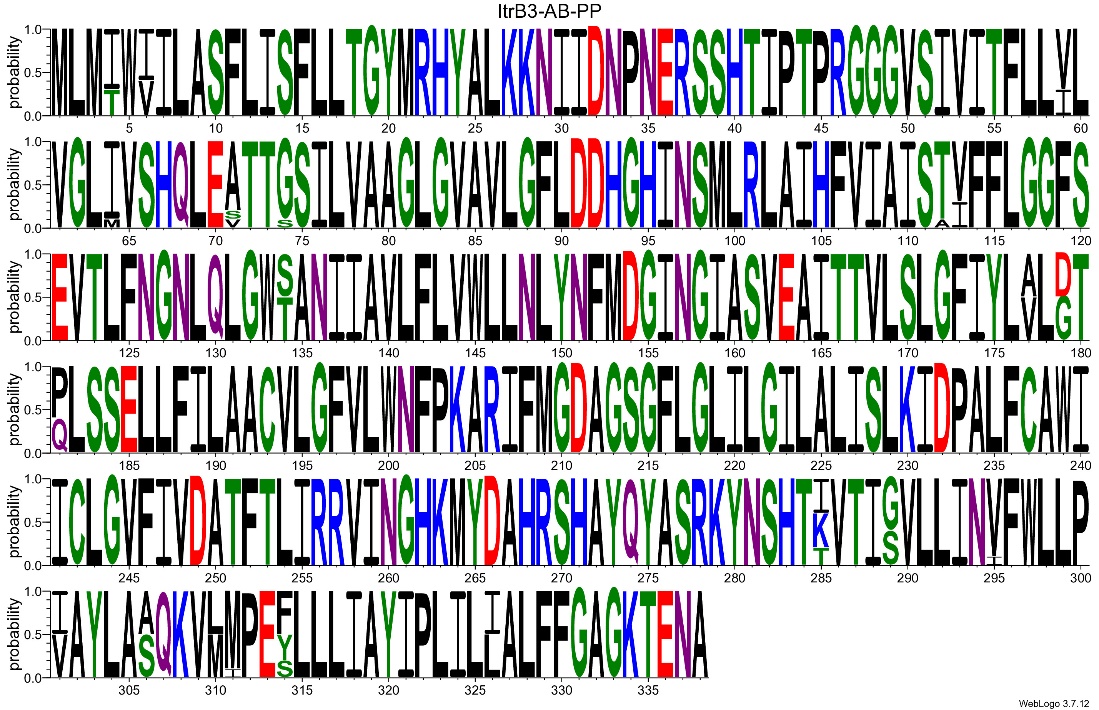** |
| --- |
| **Figure S7.** Weblogo reflecting the sequence diversity/similarity of ItrB3 among the 237 KL-types of *A. baumannii*. |

| 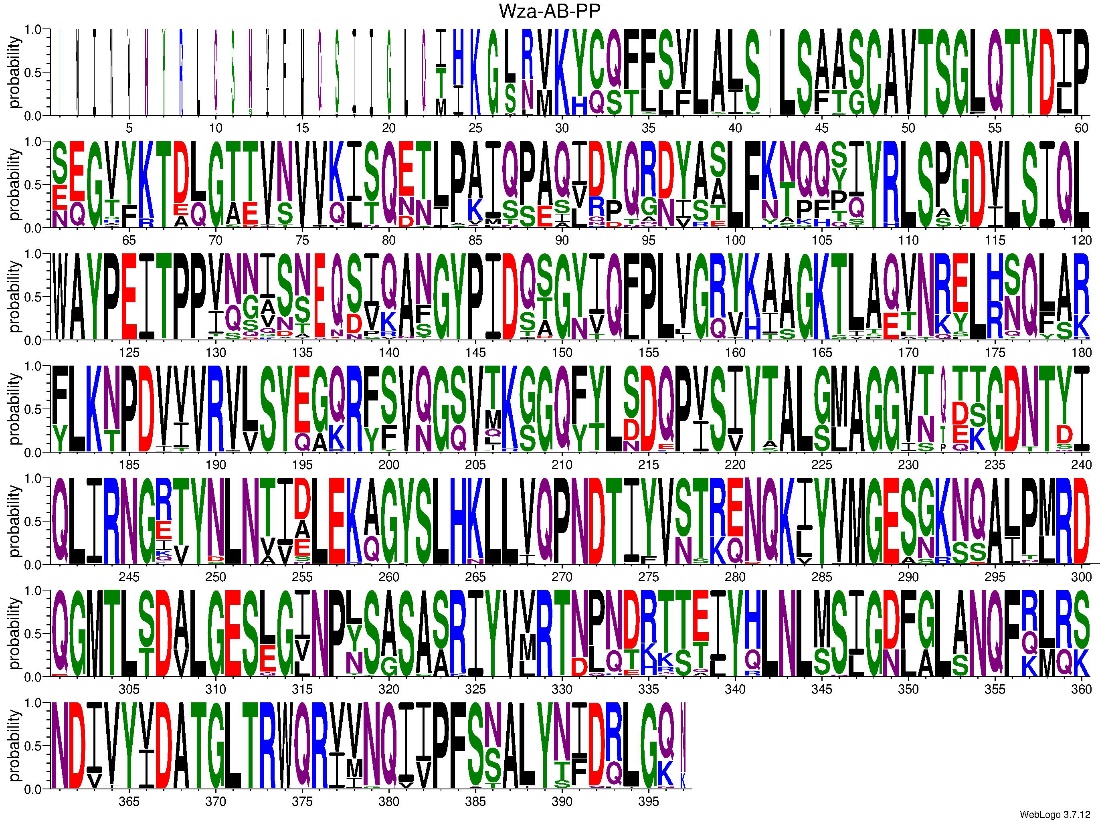 |
| --- |
| **Figure S8.** Weblogo reflecting the sequence diversity/similarity of Wza among the 237 KL-types of *A. baumannii*. |

| 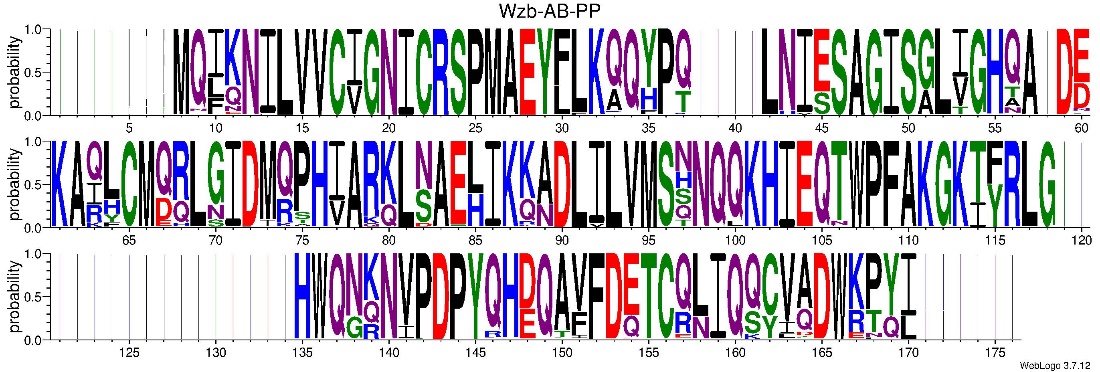 |
| --- |
| **Figure S9.** Weblogo reflecting the sequence diversity/similarity of Wzb among the 237 KL-types of *A. baumannii*. |

| 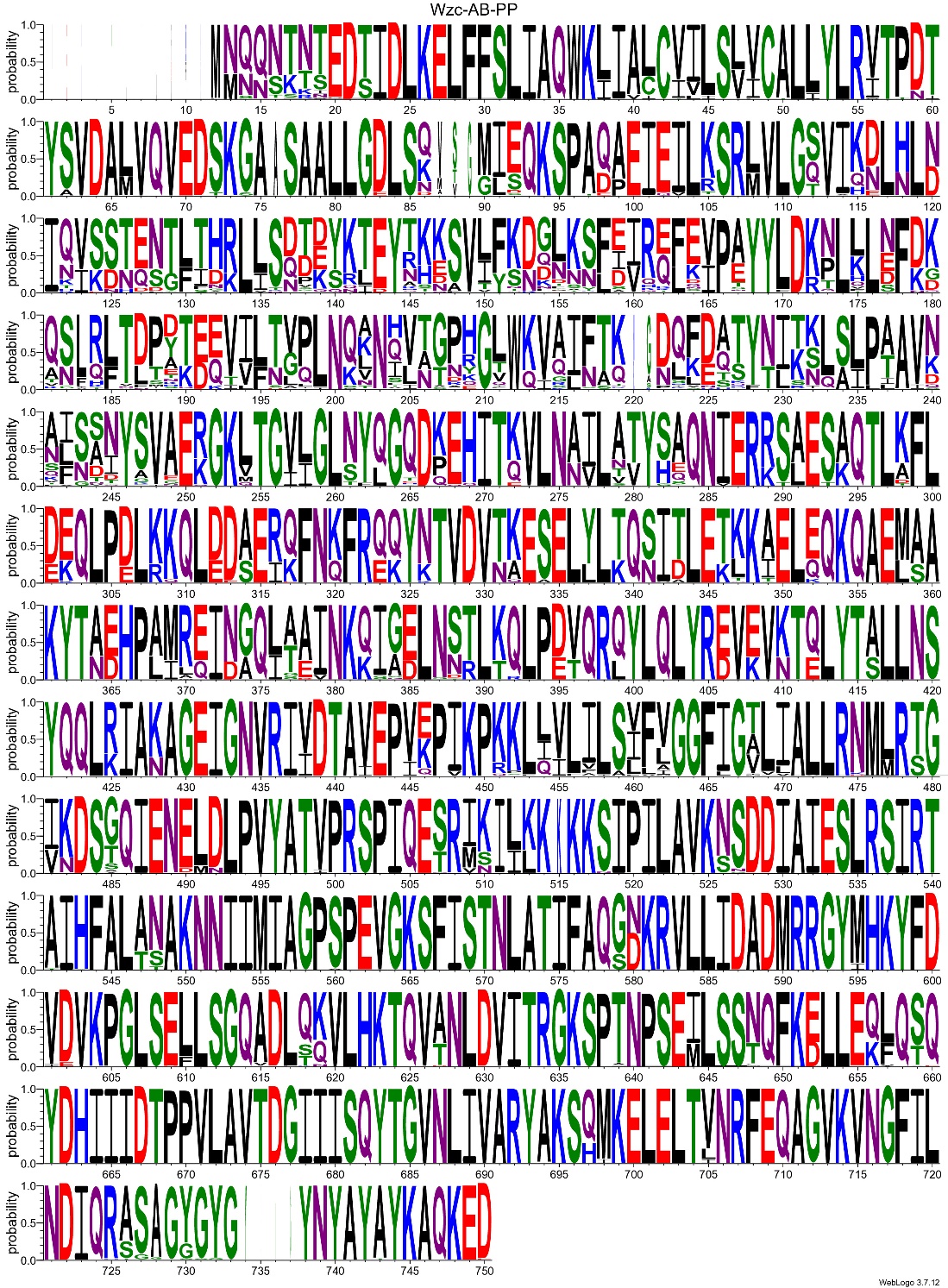 |
| --- |
| **Figure S10.** Weblogo reflecting the sequence diversity/similarity of Wzc among the 237 KL-types of *A. baumannii*. |

| 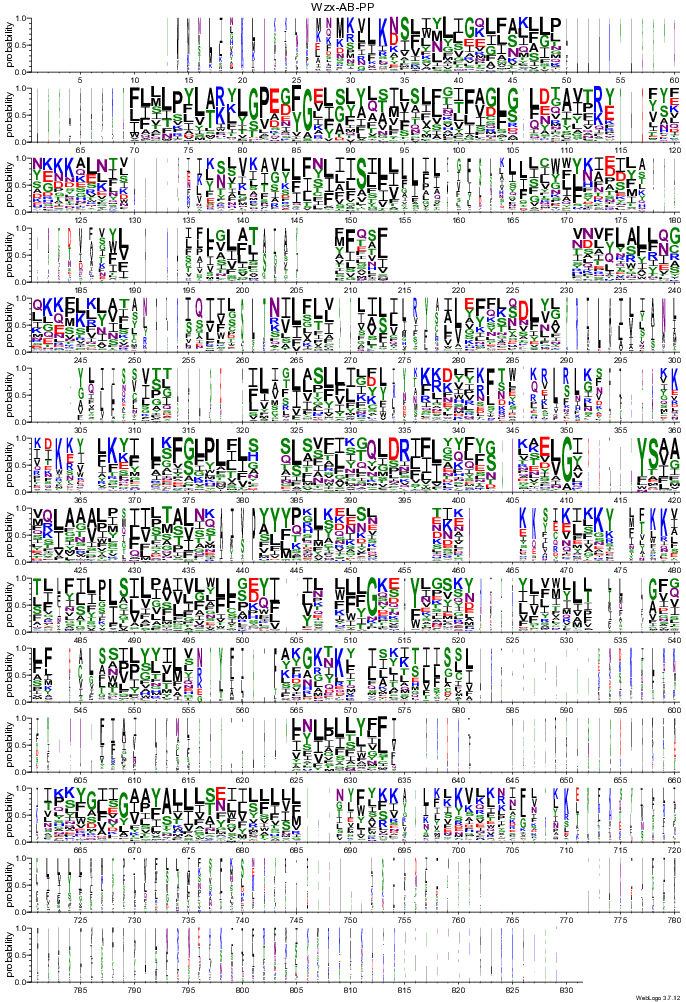 |
| --- |
| **Figure S11.** Weblogo reflecting the sequence diversity/similarity of Wzx among the 237 KL-types of *A. baumannii*. |

| 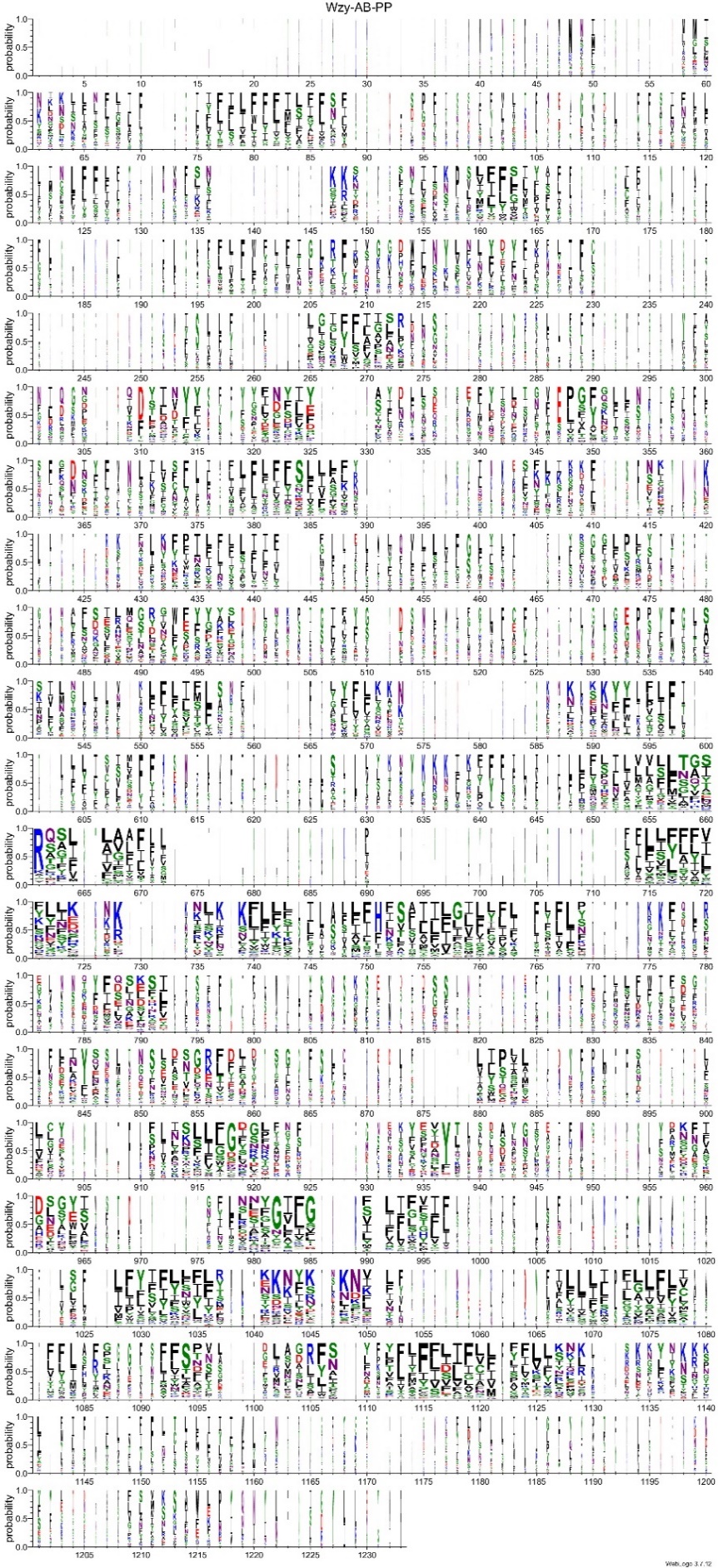 |
| --- |
| **Figure S12.** Weblogo reflecting the sequence diversity/similarity of Wzy among the 237 KL-types of *A. baumannii*. |

| 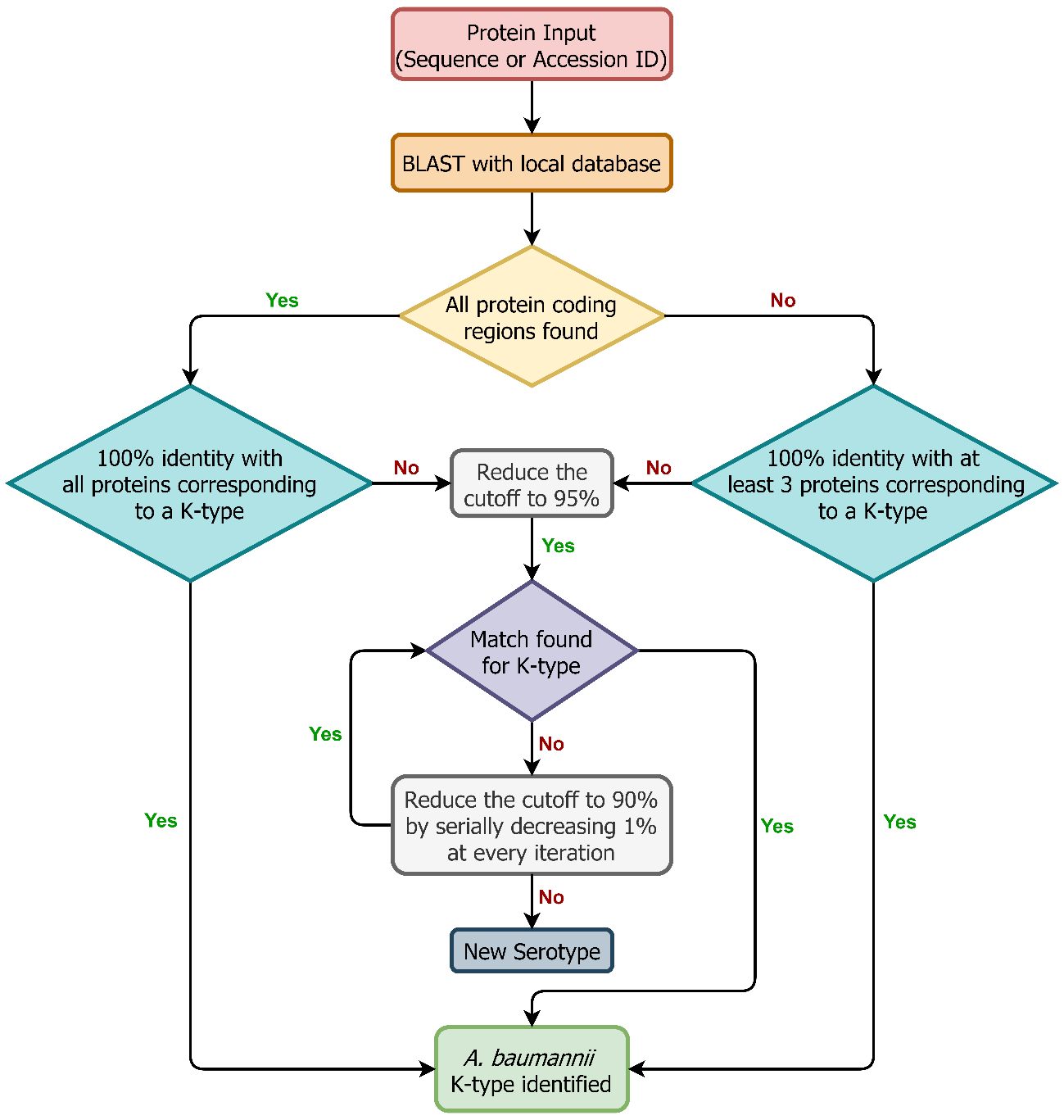 |
| --- |
| **Figure S13.** Flowchart describing the methodology followed to predict the K-type of *A. baumannii*. |

| **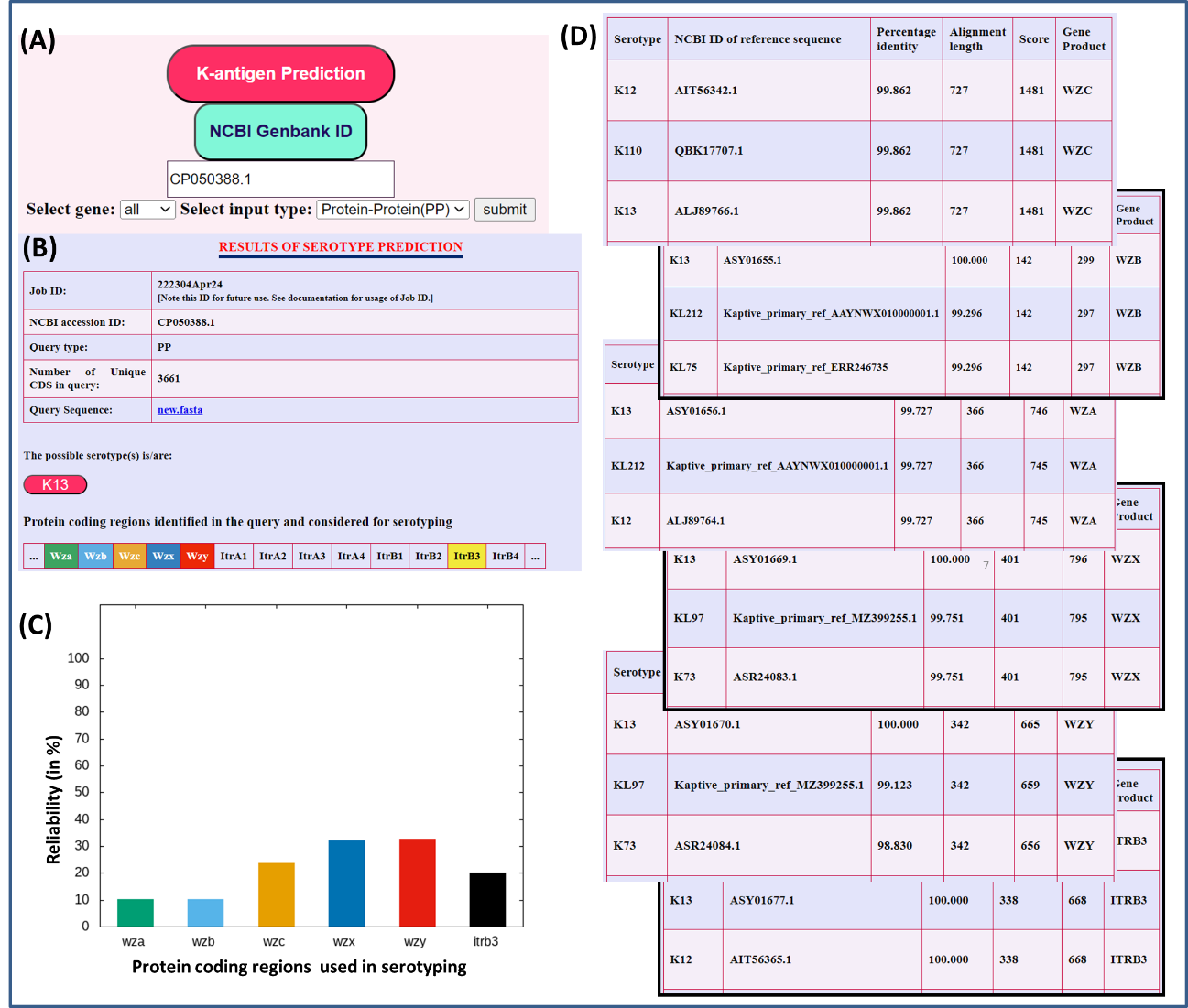** |
| --- |
| **Figure S14.** K-type prediction for *A. baumannii* strain D36 from its complete genome sequence (NCBI ID: CP050388.1). (A) to (D) shows the serotype prediction results obtained using the "Serotype predictor" module of the ABSD web tool. Note that the tables shown in (D) are the overlayed fragments of the tables (top portion) displayed on the result page of the module. |

| **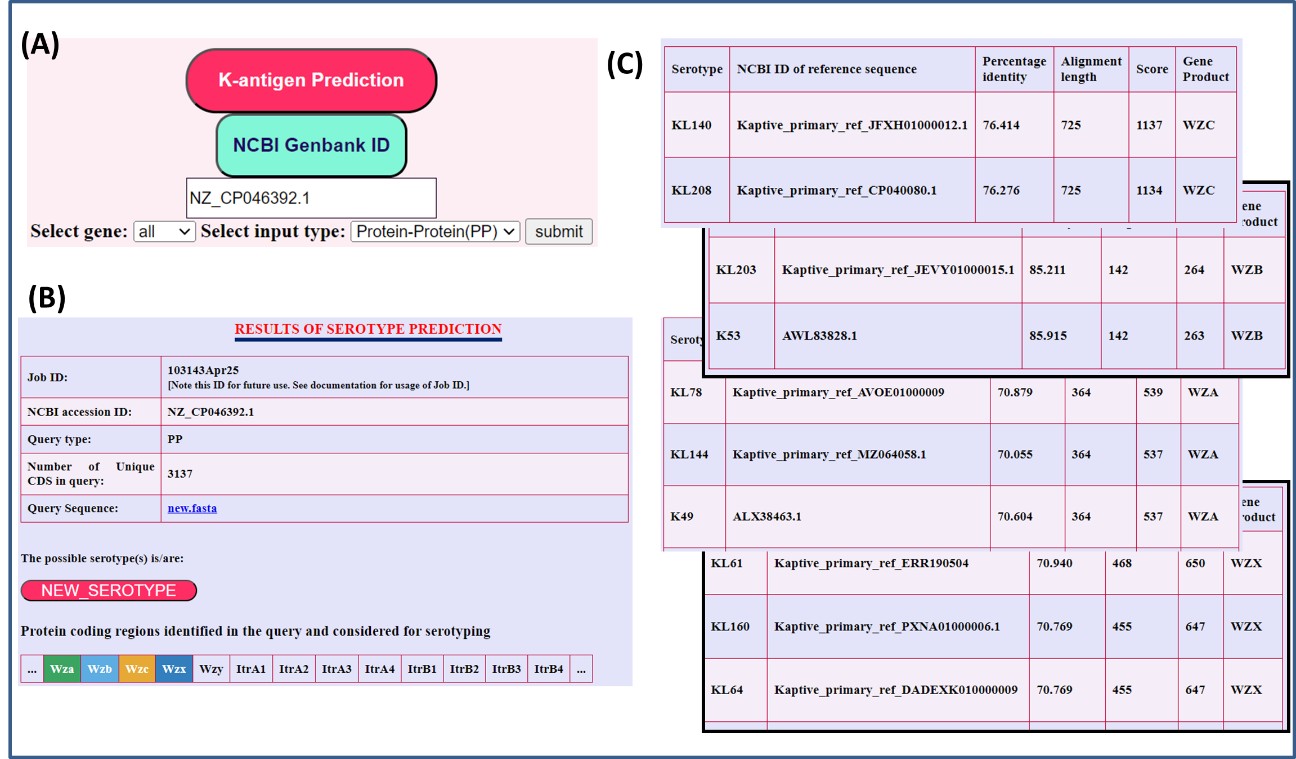** |
| --- |
| **Figure S15.** Illustration of ABSD for a new serotype prediction. (A) *Acinetobacter indicus* strain WMB-7 complete genome sequence (NCBI ID: NZ_CP046392.1) is taken as an example. (B) and (C) show the serotype prediction results obtained using the "Serotype predictor" module of the ABSD web tool. Note that there are no reference sequences corresponding to *Acinetobacter indicus* species in the current version of ABSD. Although Wza, Wzb, Wzc and Wzx sequences are identified in the input query, due to the less similarity with the reference sequences as well as the absence of Wzy and Itrs, the serotype is predicted as a "New Serotype". |
